# From imaging to precision: low cost and accurate determination of stereotactic coordinates for brain surgery *Sapajus apella* using MRI

**DOI:** 10.3389/fnins.2024.1324669

**Published:** 2024-02-01

**Authors:** Laís Resque Russo Pedrosa, Leon C. P. Leal, José Augusto P. C. Muniz, Caio de Oliveira Bastos, Bruno D. Gomes, Lane V. Krejcová

**Affiliations:** ^1^Institute of Biological Sciences, Federal University of Pará, Belém, Brazil; ^2^National Primate Center, Institute Evandro Chagas, Ananindeua, Brazil

**Keywords:** non-human primates, magnetic resonance imaging, brain, neurosurgery, stereotaxic coordinates

## Abstract

The capuchin monkey (*Sapajus apella*), a New World monkey species, exhibits prominent characteristics that make it an ideal model for neuroscience research. These characteristics include its phylogenetic traits, telencephalization coefficient, anatomical structures and pathways, genetic profile, immune responses, cognitive abilities, and complex behavioral repertoires. Traditionally, methodologies for stereotactic neurosurgery in research models have relied on the use of brain atlases. However, this approach can lead to errors due to the considerable variation in brain size and shape among individual monkeys. To address this issue, we developed a protocol for deriving individual coordinates for each monkey using a straightforward and relatively inexpensive method involving MRI imaging. Our protocol utilizes a specially designed, 3D-printed stereotactic head-holder that is safe to use with an MR magnet, non-invasive placement of fiducial markers, and post-processing with open-source software. This approach enhances MRI data visualization, improves anatomical targeting, and refines the design of neurosurgical experiments. Our technique could also prove beneficial in other areas of neuroscience research that require accurate calculation of stereotaxic coordinates. Furthermore, it could be useful for other nonhuman primate species for which brain atlases are typically unavailable.

## 1 Introduction

The refinement of neurosurgery techniques for the manipulation of cortical and subcortical targets is crucial for obtaining reliable and reproducible results. This is fundamental to neurology and neuroscience research. Non-human primates (NHPs) serve as invaluable animal models for predicting accurate preclinical results in biomedical research. Despite representing only a small proportion of vertebrate animals, many species are utilized in neuroscience research. Their value as predictive models are further enhanced due to their sophisticated behavior, and the anatomical and functional similarities of their nervous systems to humans (Chudasama and Robbins, 2006; Arnason and Clausen, 2016) (see Table 1).

**Table 1:** Comparison between different species of non-human primates used in neuroscience research.

	*Callithrix jacchus*	*Sapajus apella*	*Macaca Mulatta*	*Chlorocebus aethiops*	*Macaca fuscata*
General Biologic Characteristics	Small body size (~400 g)	Larger body size (~4 kg)	Larger body size (~7 kg)	Larger body size (~8 kg)	Larger body size (~11 kg)
	Rapid development (~12–15 months to sexual maturity)	Slower development (~ 5-7 yr. to sexual maturity)	Slower development (~ 4-7 yr. to sexual maturity)	Slower development (~ 5 yr. to sexual maturity)	Slower development (~ 4-7 yr. to sexual maturity)
	Old age reached early (~8 yr)	*	*	*	*
	Short-lived (~5–7 yr. in captivity, with a maximum of 16–17 years)	Longer-lived (40–50 years in captivity)	Longer-lived (~27 years in captivity)	Longer-lived (13–30 years in captivity)	Longer-lived (~27 years in captivity)
	Twin births common	Single births common	Single births common	Single births common	Single births common
	Short gestation (~4.5 months)	Longer gestation (6 months)	Longer gestation (6 months)	Longer gestation (6 months)	Longer gestation (6 months)
	Short inter-litter interval (~5–7 months)	Longer inter-litter interval (~21–24 months)	Longer inter-litter interval (~12-months)	Longer inter-litter interval (~9-months)	Longer inter-litter interval (~12-months)
	Greater phylogenetic distance to humans	Smaller phylogenetic distance to humans	Smaller phylogenetic distance to humans	Smaller phylogenetic distance to humans	Smaller phylogenetic distance to humans
Morphofunctional characteristics of the nervous system	Absolutely and relatively small, unconvoluted brains (among the smallest relative to body size)	Absolutely and relatively larger, convoluted brains	Absolutely and relatively larger, convoluted brains	Absolutely and relatively larger, convoluted brains	Absolutely and relatively larger, convoluted brains
	Higher-order parietal, temporal and prefrontal areas	Higher-order parietal, temporal and prefrontal areas	Higher-order parietal, temporal and prefrontal areas	Higher-order parietal, temporal and prefrontal areas	Higher-order parietal, temporal and prefrontal areas
	Lack of monosynaptic projections of corticospinal neurons onto the motor neurons of the spinal cord ventral horn	Presence of monosynaptic projections of corticospinal neurons onto the motor neurons of the spinal cord ventral horn	Presence of monosynaptic projections of corticospinal neurons onto the motor neurons of the spinal cord ventral horn	Presence of monosynaptic projections of corticospinal neurons onto the motor neurons of the spinal cord ventral horn	Presence of monosynaptic projections of corticospinal neurons onto the motor neurons of the spinal cord ventral horn

Stereotactic neurosurgery methodologies and neurophysiological microelectrode recordings in neuroscience research typically rely on brain atlases. These atlases are based on the assumption that morphological features are consistent across individuals relative to cranial landmarks such as the bregma, interaural line, and infra-orbital ridges. Using a stereotactic device, the atlas is created from a few subjects’ *ex-vivo* brain histology data to map and target specific brain regions by coordinates (Palazzi et al., 2008; Hardman and Ashwell, 2012). This approach is quite successful in rodents due to low intersubject variability, which across species, is reportedly less than 1 mm (Palazzi et al., 2008; Paxinos and Franklin, 2019). However, in NHPs, the variability of brain volumes is approximately 5-fold larger than in laboratory rodents and can be largely attributed to high intersubject variability (Ose et al., 2022). This significant variability in brain morphology and size presents a unique challenge for the use of brain atlases (Pereira-Pedro et al., 2017; Sansalone et al., 2020). Given the considerable variability exhibited within species, quantitative 3D morphometric analysis showed robust results in geometric allometry brain size and shape variation not associated with size in two New World Monkeys genera. Therefore, it may be inappropriate to use mean values to represent an entire species (Marroig, 2007).

Moreover, brain atlases for NHPs are largely available for the species most commonly used in neuroscience research, such as Rhesus monkeys (*Macaca mulatta*), Cynomolgus (*Macaca fascicularis*), and Marmosets (*Callitrix jacchus*). However, there are scarce references for species that are not among these, such as the robust capuchin monkeys (*Sapajus apella*), New World species with prominent characteristics for the study of complex neurological functions (Muniz et al., 2021). Species of the genus *Sapajus* have been successfully used as good models to elucidate cortical and subcortical anatomical connections (Sousa et al., 1991; Rosa et al., 1993; Adams et al., 2000). To the best of our knowledge there is only one atlas available for *Sapajus apella*, published in 1968 (Manocha et al., 1968). At that time, the genus Cebus comprised only four species: *Cebus albifrons*, *Cebus olivaceus*, *Cebus capucinus*, and *Cebus apella*. These were later separated into two distinct clades based on evidence from morphological, phylogenetic, and biogeographic studies, giving rise to two genera: the gracile (untufted) capuchins, representing the genus Cebus, and the robust (tufted) capuchins, representing the genus *Sapajus* (Alfaro et al., 2012).

Robust capuchin monkeys *(Sapajus apella)* are found in South America and are endemic to the Amazonian region. They represent one of the most widespread primates in the Neotropics. They are not an endangered species and breed well in captivity, easily adapting to human contact (Ryland et al., 1997). Although not traditionally used in neuroscience research, they serve as excellent experimental models due to their ease of handling, adaptability, and reproducibility (Table 2). They exhibit intricated neuronal circuitry, a high encephalization rate, a strong tendency toward exploitation and manipulation, complex social behaviors, and cognitive abilities, such as tool usage (Visalberghi, 1993). These characteristics, along with other cost–benefit related factors, make them a very interesting research model for both basic and applied studies of the nervous system (Muniz et al., 2021).

**Table 2:** The number of articles using *Sapajus apella* as an animal model in neuroscience research per decade.

Species	Number of articles (2003–2013)	Number of articles (2014–2023)
*Sapajus apella*	21	48
*Callithrix jacchus*	117	422
*Macaca mulatta*	1,181	2,190
*Chlorocebus aethiops*	527	509
*Macaca fuscata*	11	50

For details, see Supplementary material S1.

To address the limitations of neurosurgical procedures for NHP species that are not often used in research, we present a low-cost and easily attainable method in this study. This method uses Magnetic Resonance Imaging (MRI) to accurately determine the precise coordinates of brain areas in *Sapajus apella*. The MRI sessions targeted subcortical areas, but this approach can be applied to a range of procedures that require stereotactic localization of both cortical and subcortical structures in different NHP species. The methodologies and technologies we have developed are highly pertinent in regions where resources are more constrained. Our protocol leverages locally available resources and is tailored to the specific ecological and socio-economic context of Brazil.

## 2 Materials and methods

### 2.1 Animal handling and ethics

The participants were seven adult male capuchin monkeys (*Sapajus apella*) from a colony at the National Primates Center primate facilities in Ananindeua, Pará, Brazil. The animals were 21.14 ± 5.17 years old and weighed 4.3 ± 0.99 kg (Table 3). They were housed in pairs in standard cages, each measuring 2.5 × 2.0 × 2.5 m. Each cage had a communication window that allowed free movement in a total space of 2.5 × 4.0 × 2.5 m. The cages were regularly enriched with toys and objects to facilitate climbing and were cleaned regularly. The lighting followed a regular day/night cycle provided by natural light (latitude 01° 21′ 56” S; longitude 48° 22′ 20″). The animals’ diet consisted of specific chow for laboratory animals, specifically for non-human primates, and was offered daily. Fruits and natural juice were also part of the diet. Water was available *ad libitum*. All procedures were performed according to the guidelines of Directive 2010/63/EU of the European Union and under the approval of the Ethics Committee for the Use of Animals from the Evandro Chagas Institute (CEUA/IEC), protocol numbers 45/2016 and 37/2018.

**Table 3:** Characteristics of the experimental subjects.

Subject	Sex	Age	Weight (Kg)
AM-BEM	M	18	4.130
AM-BEG	M	18	3.914
AM-AOR	M	28	4.288
AM-ASA	M	28	4.362
AM-AXD	M	23	4.362
AM-BBH	M	17	3.210
AM-BCL	M	16	3.536

### 2.2 Preparation for imaging procedures

**Figure 1:**
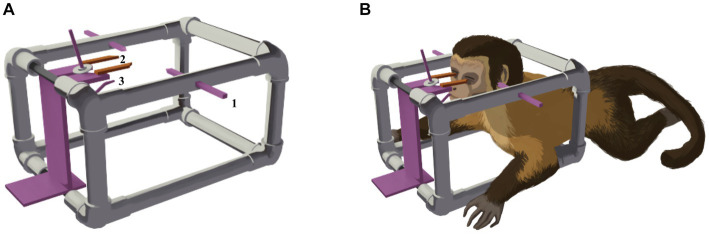
**(A)** Diagram of the 3D-printed, non-metallic stereotactic apparatus. The ear bars (1), orbital bars (2), and mouth adaptor (3) are similar to those used in the surgical apparatus. **(B)** Schematic drawing of the animal positioned in the apparatus for image acquisition. The ear bars are positioned in the right and left external acoustic meatus, the eyepieces are on the right and left inferior orbital margins, and the mouth adaptor is securely fixed to the palatus.

**Figure 2:**
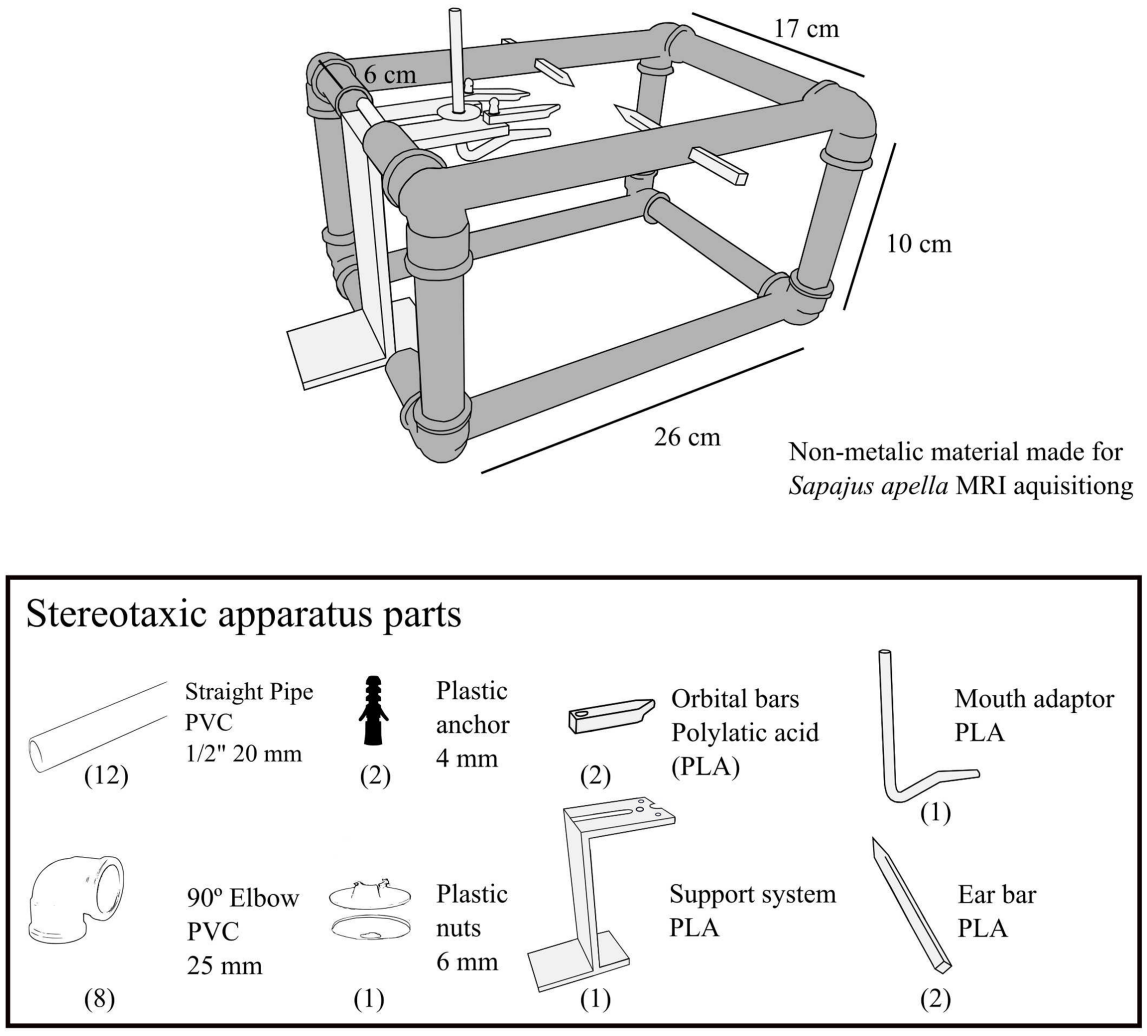
Non-metallic apparatus for MRI acquisition. The frame, depicted in dark gray, was constructed from utility engineering materials, including PVC pipes and elbows. The components in lighter gray were produced using a Sethi3D S3 printer, which employs Fused Deposition Modeling (FDM) technology. Polylactic acid (PLA) was chosen for printing due to its high resolution, structural rigidity, and minimal shrinkage. The 3D models of each component can be accessed in the. STL file (refer to Supplementary material S2). Assembly was made easy by straightforward hardware fittings. The alignment of the stereotaxic device was executed with precision using a square and level.

**Figure 3:**
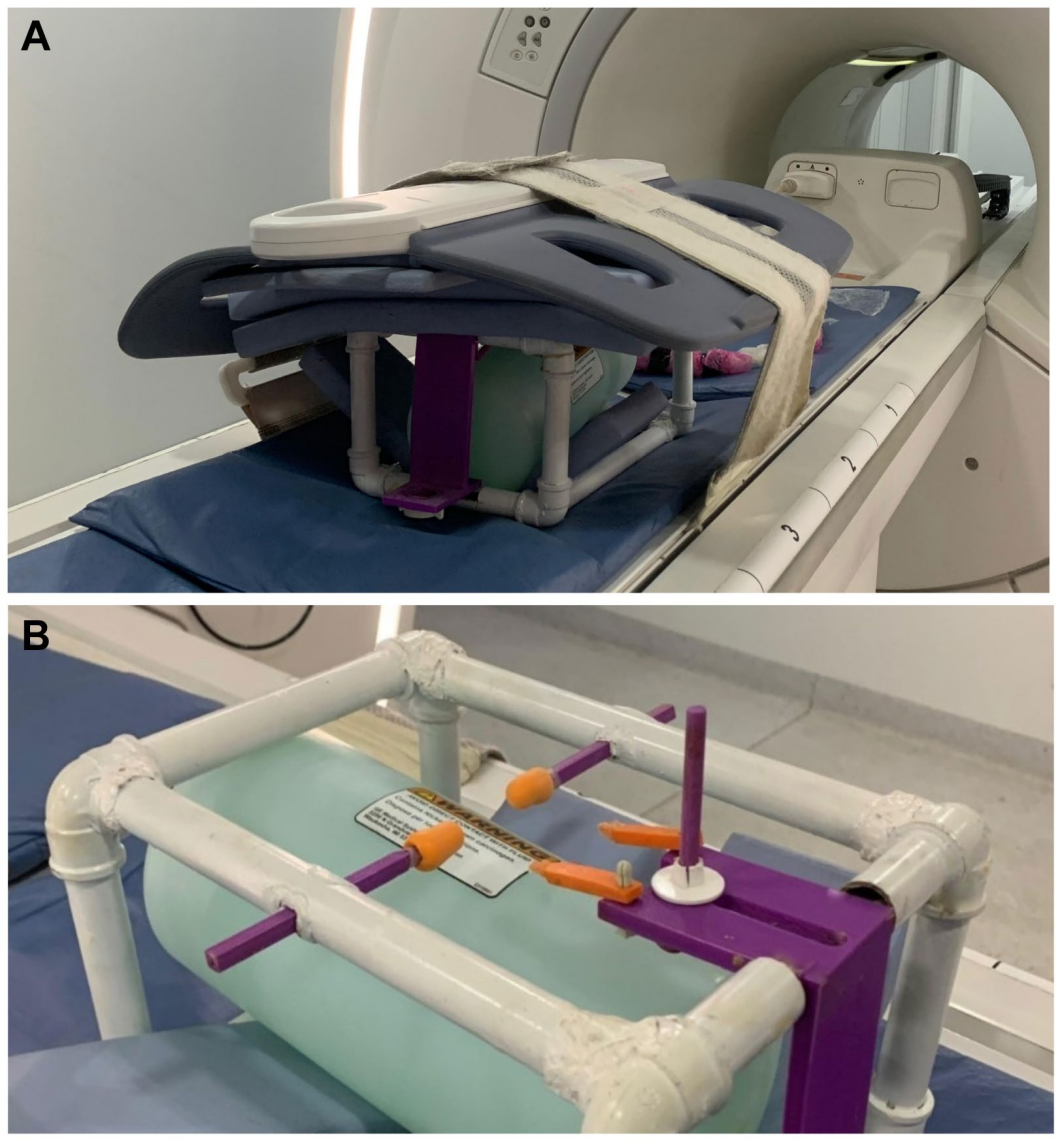
**(A)** This image showcases the stereotactic apparatus equipped with a standard receive coil for MRI examinations. For this study, a phantom was used to enhance the control and precision of the image acquisition. **(B)** Close-up of the stereotaxic apparatus with a phantom near the ears and orbital bars coated with industrial silicone.

The imaging procedures were performed with the animals anesthetized by using 9 mg/kg of pethidine and 1.2 mg/kg of midazolam, followed by propofol at 2 mg/kg maintained by infusion at the rate of 0.4 mg/kg/min (Galante et al., 2019). The animals were transported to the imaging facilities in portable stainless steel containment cages (0.80 m × 0.90 m × 0.80 m), with constant anesthetic monitoring by a veterinarian and two handling technicians. For proper positioning of the animals to enter the MRI machine, they were placed in a non-metallic (polylactic acid plastic/PLA) stereotactic apparatus (a 3D printed copy of the original stereotactic apparatus—Narishige^®^, see Figures 1, 2 for some details). Correct placement of ear bars into the acoustic meatus, and placement of the mouth adaptor and orbital bars were performed to guarantee alignment of the orbitomeatal plane. Small fish oil capsules were glued to the head at the locations of the infraorbital foramen, and the tip of each ear bar was coated with cotton embedded in a fish oil solution, to increase signal in these areas and facilitate further localization. The animal in the apparatus was then positioned on an MRI bed-sled that fit into the bore of the MRI machine. An 8-channel receive coil was placed over this stereotaxic system (see Figure 3A). To enhance flexibility and ensure animal safety, the orbital bars and the edges of the ear bar were coated with industrial silicone (Figure 3B). After the image acquisition, the anesthetic supply was suspended, and the animal was monitored until it fully recovered.

### 2.3 Assessment of MRI compatibility

To ensure that the stereotactic device does not cause image loss, an MRI was also acquired using a phantom framed and frameless. Phantom volumes were calculated using rendering and parameters calculations of SLICER 3D^©^ software (see Supplementary material S3). To define the SNR (Signal-to-Noise Ratio) values of each image, the average signal intensity was calculated and then divided by the standard deviation of noise reference. The average signal intensity was measured using ROI with a 10-point square in the center of each image. The reference noise, ROI with a 10-point square in the top left corner of each image.

### 2.4 Image acquisition and data processing

**Figure 4:**
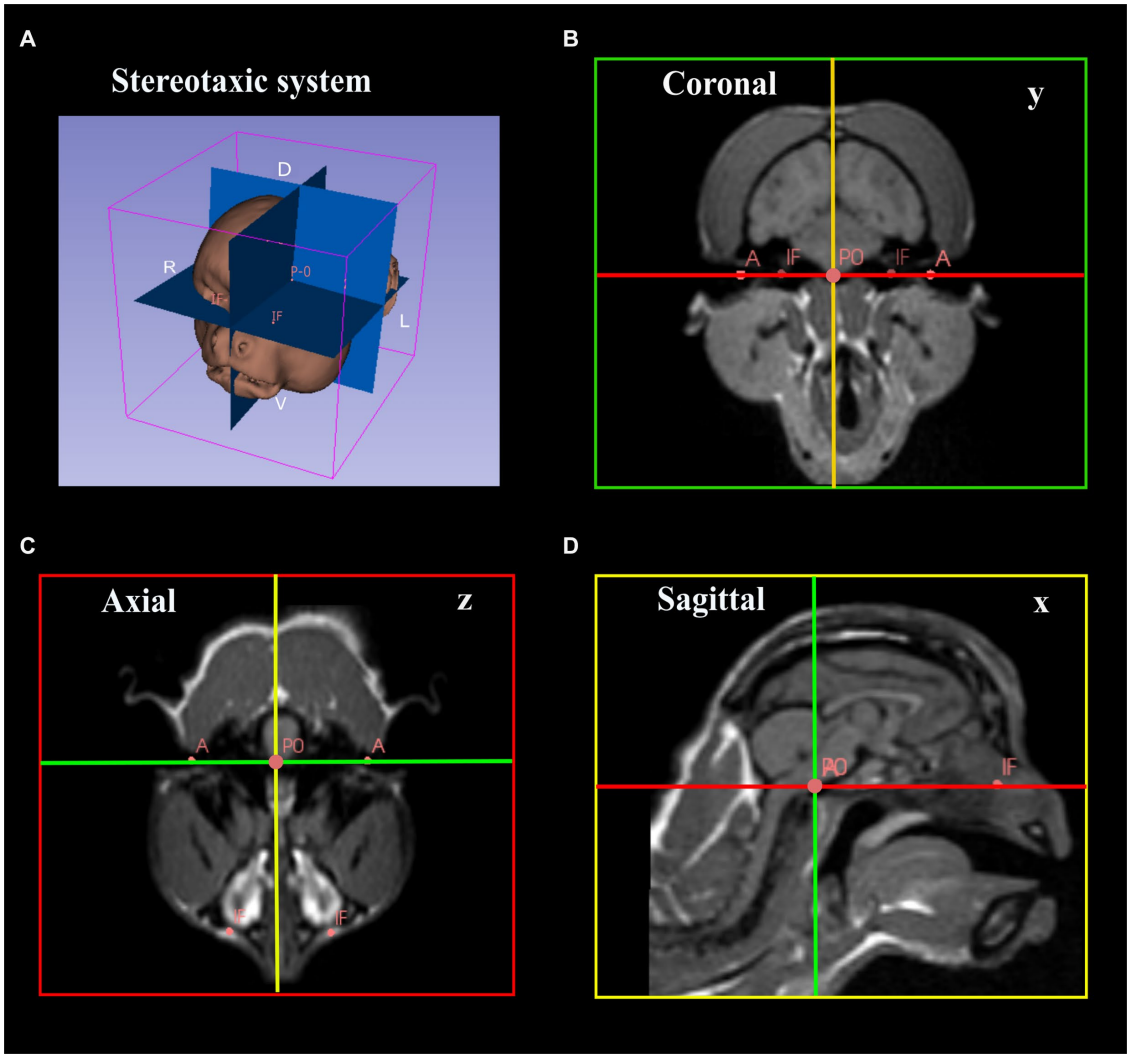
Image data processing for determination of stereotactic coordinates. **(A)** Image rendering and placement of the reference planes. **(B–D)** Placement of anatomical and fiducial markers and alignment of the coronal, axial, and sagittal planned perpendicularly and aligned with the reference points. **(B)** Coronal image passing through the intermeatus line (green line, y). **(C)** Axial image passing through the transverse axis (red line, z). **(D)** Sagittal image passing through the longitudinal fissure (yellow line, x) (A, Accustic meatus; IF, infraorbital foramen; P0, the origin).

MR anatomical images were acquired in a SIGNA^™^ Creator 1,5 T using a 3D T1 Cube (voxel/pixel ratio = 1). Whole-brain images were acquired in a 3D volume with a 256x256x256mm matrix; a slice thickness of 0.6 mm, NEX = 1, TR = 500 ms, and a minimum echo time. Images were uploaded in DICOM (Digital Imaging and Communications in Medicine) format. Post-processing was performed in SLICER 3D^©^ software. This software provides a tridimensional reconstruction of the animal’s brain through MRI sections uploaded in DICOM files. These reconstructions were used for brain volume rendering and calculation, followed by the placement of anatomical (infraorbital foramina, acoustic meatus) and fiducial markers (middle point of the interaural line) that were used as references for aligning the stereotactic planes. For a step-by-step description of the procedures on the software, see Supplementary material S3. The anteroposterior zero plane (AP-0) was represented by a straight line passing through the width of the brain at the center of the external auditory meatus. The origin (zero point) of the stereotactic coordinates corresponded to the midpoint between the two ear-bars, with the horizontal plane parallel to the orbitomeatal (OM) plane as determined by the two ear-bars and two eye-bars. The axial, coronal, and sagittal planes were then aligned perpendicularly at the zero point (Figure 4).

### 2.5 Determination of the stereotaxic coordinates

**Figure 5:**
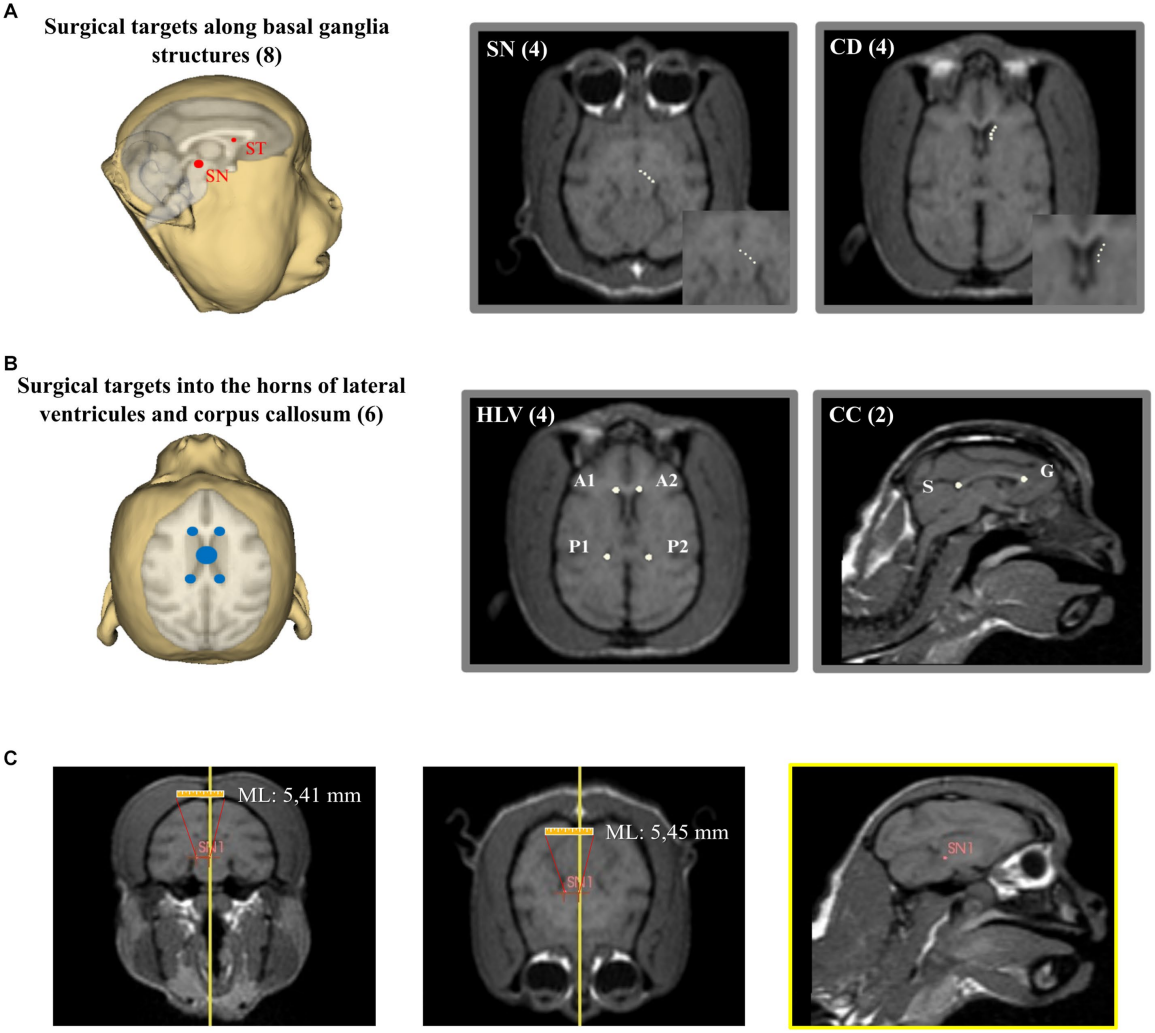
Determination of the stereotaxic coordinates. **(A)** Placement of surgical targets through basal ganglia structures (SN, substantia nigra; CD, caudate nucleus). **(B)** Placement of surgical targets through lateral ventricles and corpus callosum (HLV, horns of lateral ventricles; CC, corpus callosum; S, splenium; G, genus). **(C)** Visual confirmation of coordinates determination. In this case, SN1 coordinate was manually measured as 5,45 mm in medio-lateral (ML) which confirms the surgical coordinates previously calculated.

After the image rendering and placement of the reference planes, 14 potential surgical targets were placed in easily evident and well-defined brain structures: corpus callosum (genus – G, and splenium – S), anterior horn of lateral ventricles (left – A1 and right – A2), posterior horn of the lateral ventricles (left – P1 and right – P2), substantia nigra (four points distributed laterally through the nucleus length – SN1-4) and caudate nucleus (four points distributed anteroposteriorly through the nucleus length – C1-4) (Figures 5A,B). The coordinates of each target were calculated automatically by the 3D Slicer software. All coordinates calculated by the program were visually confirmed by two experimenters who measured the target points manually using the software’s rule (Figure 5C).

### 2.6 Statistical analysis

We performed statistical analysis using GraphPad Prism1 8.0 Software. First, we tested the normality (Kolmogorov–Smirnov) and homogeneity (Levine test) of the data. A parametric statistical analysis using ANOVA (One-way, α = 0.05, Tukey post-test) was performed for each axis (AP, DV, ML) to detect differences between coordinates. Pearson correlation coefficients were calculated to verify possible correlations between stereotaxic coordinates, brain volumes, and cephalic perimeters. The coefficient of variation was calculated for axis coordinates and brain volumes. The confidence interval was set to 95% (*p* < 0.05).

For MRI compatibility comparisons, we tested the normality test (Shapiro–Wilk). A non-parametric statistical analysis using the Mann–Whitney test (U, α = 0.05) was performed for SNRs values to detect any device susceptibility on MRI acquisition.

## 3 Results

### 3.1 Assessment of MRI compatibility

**Figure 6:**
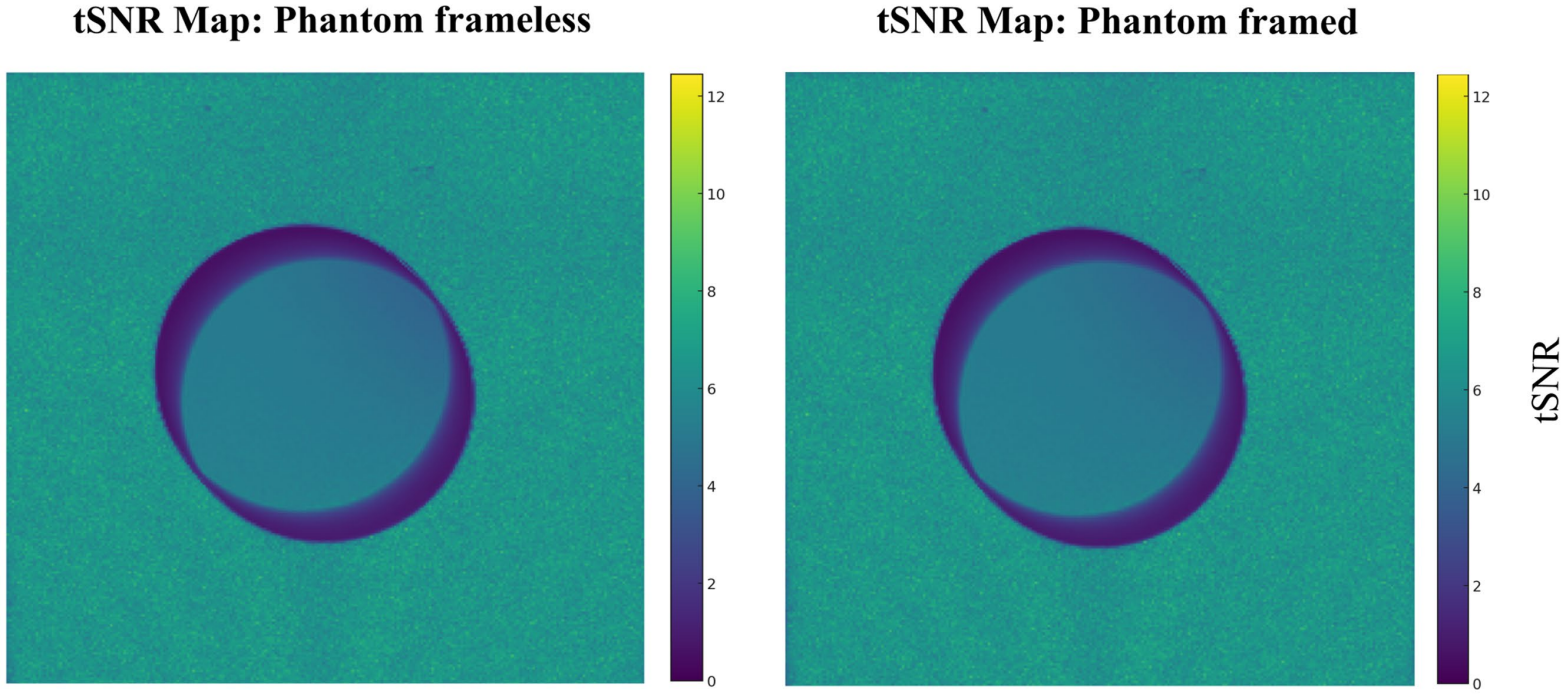
Temporal SNR for phantom frameless, and phantom framed. The same protocol was used for both conditions.

The same MRI acquisition protocol was conducted with a phantom in the stereotactic system and solely phantom to assess MRI compatibility comparisons (Table 4 and Supplementary material S1). There is no significant difference in SNR values (*p* = 1). This confirmed that there was no interference in MRI acquisition, which validates the use of the machined non-metallic stereotactic apparatus. Note there is no difference induced by the device (Figure 6).

**Table 4:** Volume and average SNRs for the scans using the phantom with and without the stereotactic frame.

MRI phantom	Volume (cm^3^)	Average SNRs
Phantom frameless	150.1	38
Phantom framed	150.1	38

### 3.2 Normality test

The normality test identified one of the animals (AM-AOR/Velhinho) as an outlier. Given that the MRI images taken for this animal showed a significant movement artifact, which made it difficult to place the markers and surgical targets, we excluded this subject from the sample. The results of the normality tests (Kolmogorov–Smirnov) are presented in Table 5.

**Table 5:** Normality test results for all coordinates in each of the three axes (mm).

Coordinate	Min	Max	Mean	S.D.	S.E.	KS dist.	*p*
**ANTERO-POSTERIOR**							
A1	24.42	29.96	26.69	2.055	0.8389	0.1645	>0.10
A2	24.76	28.19	26.36	1.379	0.5629	0.1791	>0.10
P1	0.577	6.645	3.217	2.076	0.8475	0.1607	>0.10
P2	0.801	5.783	2.978	1.808	0.738	0.1814	>0.10
S	0.477	4.598	1.959	1.45	0.5918	0.2526	>0.10
G	27.42	29.1	28.3	0.728	0.2972	0.2188	>0.10
SN1	4.779	9.098	6.653	1.553	0.6342	0.1792	>0.10
SN2	6.238	10.25	7.839	1.478	0.6036	0.1878	>0.10
SN3	7.537	11.55	9.047	1.521	0.621	0.2119	>0.10
SN4	8.968	12.76	10.22	1.604	0.6547	0.2606	>0.10
CD1	18.71	22.4	20.08	1.375	0.5613	0.2161	>0.10
CD2	20.09	23.66	21.74	1.437	0.5866	0.1919	>0.10
CD3	21.39	24.96	23.38	1.441	0.5883	0.1975	>0.10
CD4	22.73	26.61	24.79	1.499	0.6118	0.1591	>0.10
**DORSAL-VENTRAL**							
A1	20.83	24.27	22.58	1.331	0.5436	0.2916	>0.10
A2	20.98	24.38	22.62	1.311	0.535	0.2806	>0.10
P1	20.87	24.31	22.55	1.297	0.5293	0.2599	>0.10
P2	20.79	24.32	22.52	1.3	0.5305	0.2505	>0.10
S	17.16	22.29	19.48	1.731	0.7069	0.1945	>0.10
G	21.28	26	22.97	1.763	0.7196	0.2386	>0.10
SN1	12.61	16.59	13.93	1.47	0.5999	0.2317	>0.10
SN2	12.7	16.76	13.97	1.519	0.6199	0.2549	>0.10
SN3	12.4	16.76	13.91	1.57	0.6411	0.259	>0.10
SN4	12.25	16.75	13.88	1.584	0.6468	0.2661	>0.10
CD1	19.75	23.38	21.66	1.211	0.4944	0.1915	>0.10
CD2	19.83	23.07	21.62	1.088	0.4442	0.199	>0.10
CD3	19.84	23.32	21.66	1.165	0.4758	0.1545	>0.10
CD4	19.77	23.23	21.71	1.179	0.4814	0.22	>0.10
**MEDIO-LATERAL**							
A1	3.172	4.647	3.709	0.5062	0.2066	0.2919	>0.10
A2	2.917	4.488	3.563	0.5651	0.2307	0.2986	>0.10
P1	6.056	7.877	6.795	0.7782	0.3177	0.2489	>0.10
P2	6.66	7.129	6.901	0.1756	0.07168	0.1321	>0.10
S	1.194	2.196	1.538	0.3893	0.1589	0.1882	>0.10
G	1.185	1.771	1.315	0.2318	0.09461	0.3565	0.0165
SN1	5.236	6.375	5.64	0.5164	0.2108	0.3242	0.048
SN2	3.576	5.072	4.252	0.6186	0.2525	0.2369	>0.10
SN3	2.07	3.799	2.874	0.6853	0.2798	0.2477	>0.10
SN4	0.918	2.577	1.539	0.6629	0.2706	0.3298	0.0402
CD1	3.572	4.653	4.062	0.3827	0.1562	0.1327	>0.10
CD2	3.361	4.498	3.768	0.3882	0.1585	0.3099	0.0738
CD3	3.053	4.85	4.035	0.5943	0.2426	0.2204	>0.10
CD4	3.108	6.134	4.761	1.015	0.4145	0.1861	>0.10

### 3.3 Individual calculation of stereotaxic coordinates

The stereotactic coordinate calculations were performed individually according to the procedures described above. The individual coordinates for each of the surgical targets, and for the three anatomical axes are presented in Table 6. ANOVA tests comparing the coordinates for all animals in each target did not show significant differences between the measurements obtained for the subjects. The averages were significantly different enough to reject the null hypothesis of coordinates equality between monkeys at each axis (AP [*p* = 0.98]; DV [*p* = 0.19]; ML [*p* = 0.76]).

**Table 6:** Individual coordinate values were calculated for each surgical target in each subject (mm).

ANIMAL	AMBCL	AMAXD	AMBEN	AMBBH	AMASA	AMBEG
**ANTERO-POSTERIOR**						
A1	24.725	24.415	27.275	27.587	29.956	26.178
A2	24.762	24.959	26.840	28.193	27.471	25.954
P1	0.577	2.390	4.145	6.645	3.437	2.107
P2	0.801	1.607	4.255	5.783	2.911	2.510
S	1.728	0.869	1.826	4.598	2.254	0.477
G	27.429	27.418	28.719	29.102	28.842	28.274
SN1	4.779	5.290	6.339	9.098	7.269	7.144
SN2	6.238	6.498	7.619	10.253	8.667	7.761
SN3	7.537	7.874	8.874	11.550	10.106	8.341
SN4	9.019	9.079	9.847	12.763	11.614	8.968
CD1	19.631	19.905	22.402	18.928	20.875	18.713
CD2	21.632	21.914	23.659	20.199	22.963	20.088
CD3	23.488	23.670	24.958	21.393	24.762	21.987
CD4	24.763	25.091	26.119	22.734	26.608	23.434
**DORSAL-VENTRAL**						
A1	24.079	22.040	20.830	22.158	24.273	22.113
A2	24.038	22.040	20.981	22.244	24.380	22.059
P1	23.880	22.028	20.867	22.241	24.307	21.959
P2	23.784	22.035	20.793	22.248	24.320	21.959
S	19.857	17.155	18.595	18.877	22.294	20.094
G	23.770	23.188	21.278	21.843	26.002	21.736
SN1	14.099	13.201	12.793	12.609	16.586	14.312
SN2	14.113	13.238	12.763	12.696	16.763	14.275
SN3	14.123	13.211	12.780	12.396	16.763	14.208
SN4	14.100	13.231	12.820	12.253	16.750	14.153
CD1	22.200	21.030	19.753	21.738	23.379	21.876
CD2	22.137	21.082	19.833	21.705	23.068	21.876
CD3	22.234	21.075	19.837	21.625	23.318	21.874
CD4	22.298	21.071	19.770	22.048	23.225	21.872
**MEDIO-LATERAL**						
A1	3.762	3.172	3.628	3.655	4.647	3.392
A2	2.917	3.299	3.968	4.488	3.331	3.372
P1	6.056	6.227	6.633	6.321	7.877	7.654
P2	6.660	6.754	6.956	7.037	6.870	7.129
S	1.518	1.772	1.355	1.194	1.195	2.196
G	1.200	1.771	1.346	1.192	1.195	1.185
SN1	5.236	5.237	5.430	5.341	6.375	6.218
SN2	3.819	3.947	4.142	3.576	5.072	4.953
SN3	2.513	2.506	2.734	2.070	3.623	3.799
SN4	1.126	1.260	1.204	0.918	2.150	2.577
CD1	3.960	3.791	3.572	4.095	4.653	4.303
CD2	3.635	3.746	3.578	3.361	4.498	3.791
CD3	4.300	3.864	4.203	3.053	4.850	3.937
CD4	5.381	4.734	4.830	3.108	6.134	4.377

### 3.4 Surgical confirmation of the coordinates

**Figure 7:**
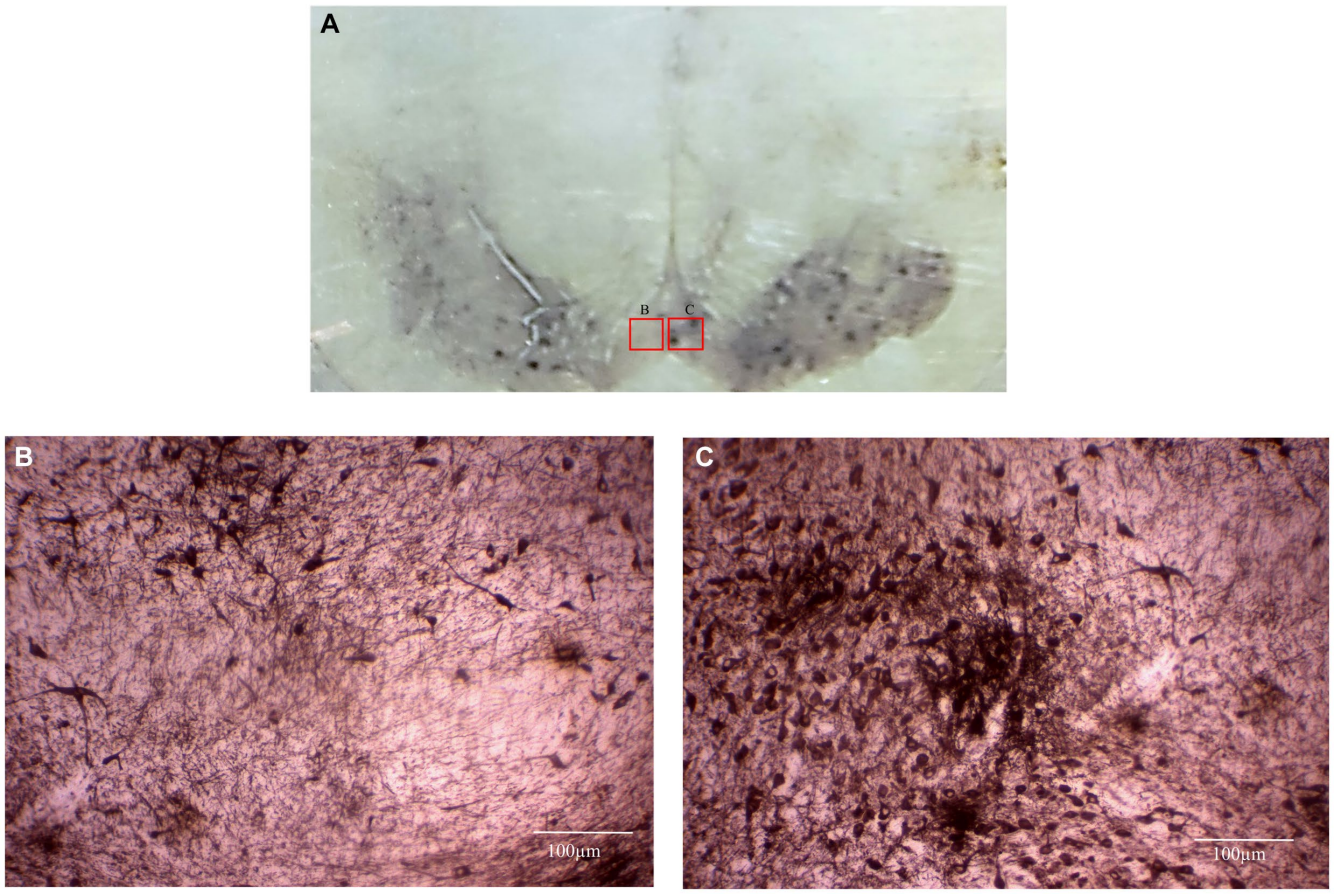
This photomicrograph depicts the mesencephalic region (substantia nigra) of a *Sapajus apella* subject, highlighting Tyrosine Hydroxylase (TH) immunohistochemistry. **(A)** At a lower magnification, the left side of the brain exhibits a lesion induced by our surgical protocol, which involved the injection of a neurotoxin. In contrast, the right side remained unlesioned, serving as a control. **(B)** A higher magnification of the area shown in Figure A indicates a notable reduction in TH-positive neurons on the lesioned left side. **(C)** A higher magnification of the unlesioned right side, as shown in Figure A, displays numerous TH-positive neurons. This data has not yet been published.

The surgical planning yielded robust results in terms of experimental accuracy. The surgical procedure, aimed at the basal ganglia, was informed by coordinates determined through MRI Slicer 3D processing and demonstrated efficiency. The conventional apparatus Narishige^®^ was used for surgical manipulation. Our surgical protocol involved unilateral injection of 6-hydroxydopamine into the substantia nigra and caudate nucleus to induce a reduction in dopaminergic neurons (see Figure 7).

## 4 Correlation analysis of brain coordinates and volumes

**Figure 8:**
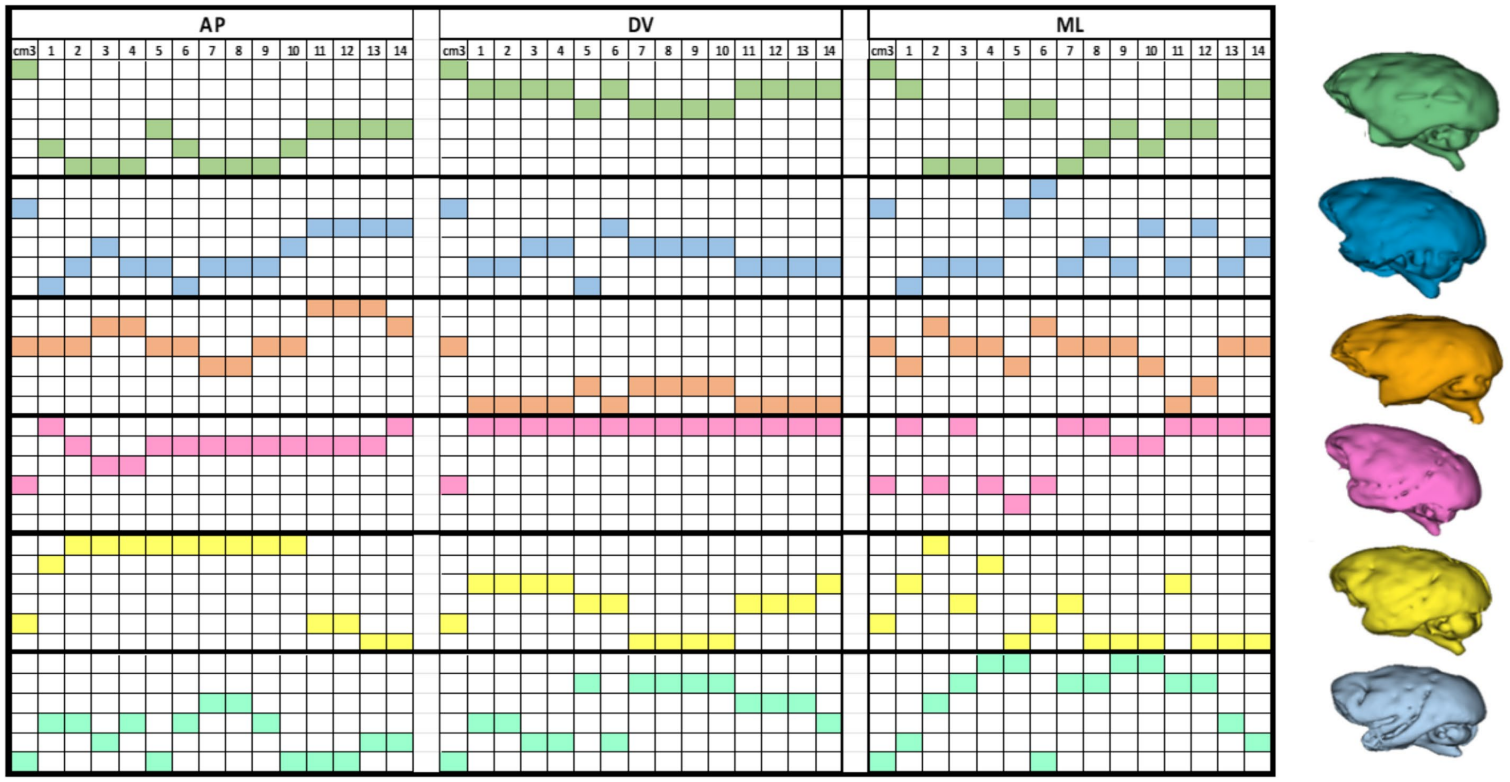
This color matrix displays individual coordinates in descending order. It is important to note that for each subject, the descending order of brain volume does not necessarily correspond to the arrangement of the coordinates in the same manner. This discrepancy reveals nonlinear variances in brain shapes, as illustrated by the 3D renderings of entire brain volumes from each MRI scan (right).

We observed no significant correlation between brain volumes and stereotactic coordinates on either axis. This suggests that the variability in coordinates does not mirror changes in overall brain volume. In other words, variations in brain volume might be indicative of nonlinear differences in skull size and shape. To demonstrate this, we arranged the data on brain volumes and coordinates in descending order and emphasized the coordinates corresponding to each animal’s brain volume. For clearer visualization, we assigned different colors to each subject’s brain, creating a coordinate matrix for each animal (see Figure 8).

### 4.1 Coefficient of variation

**Figure 9:**
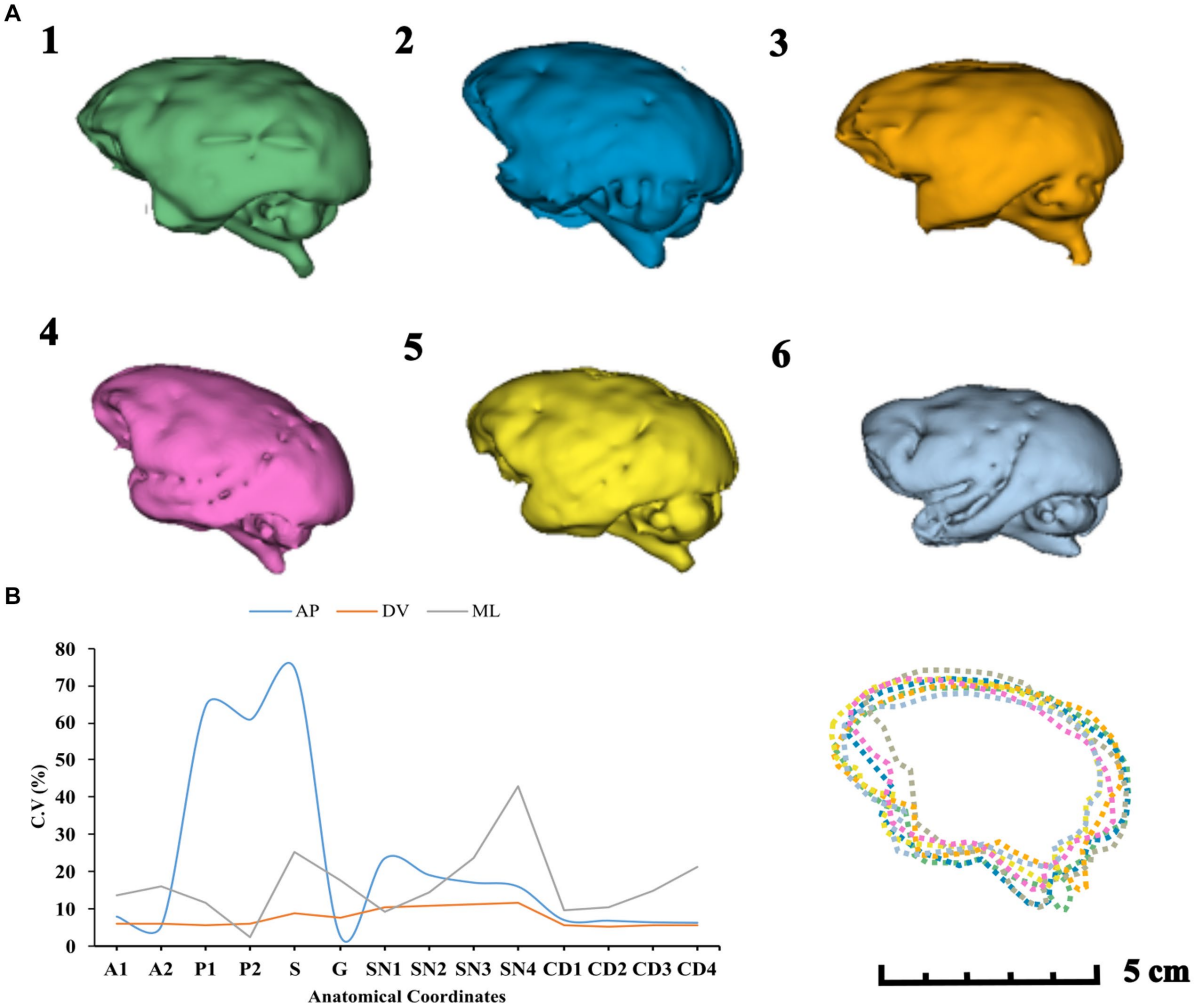
**(A)** Brain volume rendering in descending order (1–6). Brain shape overlapping comparison (7). **(B)** COV distribution of the surgical targets in anteroposterior (AP), dorsoventral (DV), and mediolateral (ML) axes.

The CV for brain volumes was 9.4%. The COV for all coordinates in different axes can be seen in Figure 9. This suggests a non-linear variation, with a higher COV value observed for AP coordinates.

## 5 Discussion

In our study, we described a technique in which MRI imaging was used to obtain stereotactic coordinates for different surgical targets in *Sapajus apella*, a gyrencephalic New World species that is a promising model for many approaches in neuroscience research. The method described here represents an important experimental refinement when compared to conventional methods (see MRI processing guide in Supplementary material S1). In primate experiments, atlas-based stereotaxy is commonly used. Errors in targeting are generally a result of either morphologic differences between the individual macaque brains, or human errors in identifying the brain landmarks (Subramanian et al., 2005). These errors in targeting and the consequent misplaced targeting are rarely reported but should be included in future publications.

Moreover, the availability of brain atlases for different species of non-human primates is scarce, and the available atlases are either entirely based on a single animal or lack adequate 3D data (Paxinos et al., 2000). As mentioned, the only brain atlas available for capuchin monkeys was published in 1968 (Manocha et al., 1968) for *Cebus apella*. At that time, the genus *Cebus* included Tufted and Untufted Capuchin monkeys, which posteriorly. These were later separated into two different genera after phylogenetic studies have confirmed a huge diversity, justifying the creation of a new taxonomic division, the genus *Sapajus* (Lynch Alfaro et al., 2012). Additionally, the aforementioned publication does not cover the integrity of the brain regions and displays only images of the basal telencephalon in the coronal plane without coverage of more superior or cortical structures.

Therefore, the need for the development of our protocol primarily stems from the lack of available stereotactic atlases for *Sapajus apella*. The literature on the use of *Sapajus apella* as an animal model for experimental neurosurgeries is quite scarce. Despite being a promising model for the study of both normal and abnormal nervous systems (Hecht et al., 2021; Muniz et al., 2021; Miss et al., 2022), capuchin monkeys are not among the most frequently used species in non-human primate biomedical research. This is likely due to practical reasons that make other NHP species more suitable for laboratory use (Bert et al., 2012; Lankau et al., 2014).

Unlike rodents, whose brain structures exhibit relatively constant size and location values, the brains of NHPs can be highly variable (Bentley et al., 2018). Primates are altricial animals, experiencing a significant postnatal brain growth spurt. Therefore, variations in environmental factors such as maternal care, food, and environmental stimuli can induce a wide range of phenotypes, reflecting on the development of the size and shape of the skull (Ikonomidou et al., 2001; De Vareilles et al., 2022) (see Table 6; Figure 8). Thus, the use of MRI for targeting significantly improves surgical accuracy as it addresses the issue of intraspecies variability in brain structure.

Based on the classical Cartesian plane, frame-based systems for neurosurgery have the advantage of proven clinical accuracy and a high degree of mechanical stability, making them the gold standard. However, there are many frameless methods available that appear to offer flexibility (Fukaya et al., 2010; Patil, 2010; Sutherland et al., 2013). Frameless systems demand greater complexity and material expenditure. In addition to requiring precise hand coordination, natural tremors may influence the error margin and surgical results (Bradac et al., 2017).

Convection-enhanced delivery (CED) is a frameless system of drug delivery to the brain through intraparenchymal microcatheters with broader achievement using fewer infusions (Bradac et al., 2017; Yazdan-Shahmorad et al., 2018). In addition to the need for compatible and expensive materials for implantation in the parenchyma, it constitutes an invasive surgical method with the potential for encephalitic states (Khateeb et al., 2019). Surgical methods must be efficient and minimize animal risk. MRI-guided surgeries are preferable to intraoperative plain film roentgenography due to associated radiation exposure issues (Damilakis et al., 2010). Beyond radiation safety, MRI-guided surgeries, as a preoperative refinement, enable virtual fiducial placement on software platforms for virtual surgical planning, which is crucial for minimizing the error rate (Farrell et al., 2014).

Our method, which uses MRI, provides superior soft tissue contrast and allows us to view the structures of the monkey brain *in vivo*. The procedure we followed for stereotactic calculations was both simple and inexpensive. Although we are aware of the existence of complex scripts and software reported in previous studies, they typically employ high-cost computational frameworks and involve several pre-processing steps (Subramanian et al., 2005; Frey et al., 2011), yet they offer similar accuracy to our method. Some protocols involve the use of fiducial markers attached to the skull or in subcortical regions, which are more invasive and can trigger unintended inflammatory responses (Asahi et al., 2003). Our method was designed to avoid invasive fixation on the stereotaxic frame when positioning the animal for MRI scanning, similar to the protocol used by Saunders (Saunders et al., 1990).

Other studies have previously applied similar protocols using Old World NHPs, primarily Rhesus (*Macaca mulatta*). For New World monkeys, the most commonly used species is the common marmoset (*Callithrix jacchus*), a New World species with a lissencephalic brain (Hikishima et al., 2011). An interesting study by Seidlitz (Seidlitz et al., 2018) provided a population-averaged MRI standard template with anatomical references for NHP brains, taking into account individual asymmetry. This included surface reconstructions and transformations to previously published digital brain atlases for Rhesus. We did not find a similar study for *Sapajus apella*.

## 6 Error margin

Although we applied a highly developed imaging method, the protocols described here are not without potential errors. We calculated a small error margin in our study by comparing the software-generated coordinates with visually controlled coordinates, which involved confirming the coordinates using the software’s manual tool. Based on these comparisons, the error margin for each coordinate in the three axes was calculated by taking the average of the differences between the software-calculated and visually-calculated coordinates, along with their standard deviation. A mean error of 0.02 ± 0.01 in the anterior–posterior, 0.09 ± 0.11 in the dorsoventral, and 0.01 ± 0.03 mm in the mediolateral was observed (see Supplementary material S1). This can occur due to factors such as MRI resolution, potential uncontrolled movement artifacts during image acquisition, and the examiner’s performance during visual control. Nonetheless, compared to the size and volume of the targeted anatomical structures, the error margin obtained here is negligible and may not be associated with a surgical error. On the other hand, the intraspecies variation observed here ranged between 4.04 ± 1.03 in the anteroposterior, 3.88 ± 0.58 in the dorsoventral, and 1.42 ± 0.62 mm in the mediolateral (see Table 4), which represents a significant distance that could lead to a targeting error in neurosurgical approaches.

### 6.1 Correlation between brain volume and stereotaxic coordinates

It is known that brain growth is accentuated during early life development. After a highly dynamic early postnatal period, brain growth stabilizes in adulthood, when interindividual differences become well established. These differences result from varying and nonlinear growth patterns in different brain structures throughout the lifespan (Coupé et al., 2017; Danielsen et al., 2020), leading to interindividual differences in brain volume, shape, development, and proportion between brain structures.

In our study, we found no correlation between total brain volume and stereotaxic coordinates, which reinforces the fact that differences in brain development are nonlinear within species. It has been shown that the same subcortical structure may exhibit different stereotaxic locations throughout the lifespan, for example, the midsagittal area of the corpus callosum. Watson et al. (2022) observed sustained growth patterns of the corpus callosum in *Sapajus apella* monkeys in the splenium and genu regions. Regional differences are apparent in these growth patterns, with the genu increasing considerably during the first 6 years of life, and the splenium showing a greater increase between seven and 18 years (Vannucci et al., 2017). Considering our sample of adult monkeys, we observed higher variations for the coordinates P1, P2, and S, the most posterior structures analyzed in this study, which may reflect the patterns reported by Watson et al. (see Figure 9).

Based on the ratio of AC–PC (anterior commissure to posterior commissure) analysis in selected groups of humans and NHPs, there are higher variances in the AP axis. The mean ± SD for AC–PC distances were 28.3 ± 1.6 mm, 12.3 ± 0.8 mm, and 13.8 ± 0.7 mm for the human, Cynomolgus, and Rhesus groups, respectively (Fiandaca et al., 2011). This correlates with our results about the higher CV in AP coordinates and also supports the difficulty of localizing deep brain targets such as striatal brain structures (Yin et al., 2009).

Morphological brain variations can be related to different environmental contexts, which involve capuchins’ extractive foraging behaviors, including motor processing and spatial ability for prey capturing, tool usage, etc. (Janson and Boinski, 1992). Furthermore, *Sapajus apella* monkeys exhibit sex differences in behavior, with differences in cortical plasticity in brain organization (Hecht et al., 2021). It has been suggested that the morphology of the corpus callosum in capuchins is influenced by sex and handedness (Phillips et al., 2007), which could be linked to differences in cortical anatomy (Phillips and Sherwood, 2008).

Previous studies have calculated coefficients of variation (COV) of brain volume for different species, having reported a higher COV of brain volume in New World monkey species than that observed in rodents: 2.3% for mice (Ma et al., 2008), 3.2% for rats (Hasegawa et al., 2010), compared to 6.6% in marmosets (Hayashi et al., 2021). The significant primate’s COV correlates with the acceptable limits of the classical stereotactic approach. Our data suggest a volume brain COV of 9,4% for *Sapajus apella* adult population.

### 6.2 Advantages and limitations

This simple, cost-effective protocol with minimal pre-processing steps for primates’ MRI-based stereotactic calculations has the potential to assist experimenters working with different animal species in refining their neurosurgical methods and improving targeting (Figure 7). The most significant advantages of our protocol lie in the ease of determining any potential brain target, both cortical and subcortical, and in considering intraspecies anatomical differences. This method, which involves using a non-metallic replica of the surgical holder during imaging and referencing anatomical landmarks, can overcome potential errors induced by anatomical asymmetries of the subjects’ heads. For instance, substantial differences in the diameters of the external auditory meatus between the right and left sides can result in a discrepancy between two coordinate systems, because the midpoint of the ear bars does not correspond to the actual interaural zero. The validation by predicting the error margin met the required safety standards, leading us to believe that our protocol will have promising applications.

We believe that an open-source paradigm is a major trend in the development of medical imaging applications. 3D Slicer has the advantage of having an intuitive work interface, an internal DICOM image convertor, and the possibility of filing results through a scene system that can make changes on previous reconstructions, besides combining different scenes to compare studies.

Despite its simplicity and consistency, there are some limitations to this alternative protocol. First, there are technical limitations such as the quality of the MRI and post-processing software tools. For instance, the manual ruler for visual control depends on the operator, and the positioning of fiducial markers relies on anatomical knowledge (Gonzalo Domínguez et al., 2016). The resolution of the MRI must be considered and can be improved by adjusting the scanning sequence and extending the scanning time. Another significant limitation lies in determining the coordinates of areas where cytoarchitectural criteria are crucial for defining boundaries. For example, many neocortical areas cannot be confidently identified by MRI imaging, which only shows the differences between gray and white matter.

## 7 Conclusion

This technique addresses the issue of significant intraspecies variability in brain size and shape, a major source of bias in atlas-based studies for stereotactic brain surgery in NHPs. Furthermore, for species that are infrequently used in neuroscience research, the lack of available brain atlases can be overcome. The use of open-source and user-friendly software for image processing makes this method cost-effective and readily accessible for use in various experimental settings. The adoption of anatomical landmarks, along with the pre-placement of the animal in a stereotactic frame for imaging, may help to avoid stereotactic errors induced by the anatomical asymmetry of the animal’s head. Additionally, preoperative MRI scanning can be beneficial in identifying preexisting brain abnormalities that meet exclusion criteria for behavioral or neuroanatomic studies. In summary, our method may be highly beneficial to areas of neuroscience research that depend on accurate stereotaxic apparatus, with minimal procedural complexity.

We hope our protocol serves as a model for similar environments, proving that significant scientific contributions can emerge from regions traditionally underrepresented in global research landscapes. This aligns with the growing recognition of the importance of contextually relevant research in developing countries, which can address local challenges and contribute to the global scientific community.

## Data availability statement

The original contributions presented in the study are included in the article/Supplementary material, further inquiries can be directed to the corresponding author.

## Ethics statement

The animal study was approved by Ethics Committee for the Use of Animals from the Evandro Chagas Institute (CEUA/IEC). The study was conducted in accordance with the local legislation and institutional requirements.

## Author contributions

LP: Conceptualization, Data curation, Investigation, Methodology, Software, Writing – original draft, Writing – review & editing. LL: Data curation, Formal analysis, Software, Writing – review & editing. JM: Conceptualization, Project administration, Writing – review & editing. CB: Data curation, Methodology, Software, Writing – review & editing. BG: Data curation, Writing – review & editing. LK: Conceptualization, Funding acquisition, Supervision, Writing – review & editing, Writing – original draft.
