# Supplementary material for: From imaging to precision: low cost and accurate determination of stereotactic coordinates for brain surgery Sapajus apella using MRI
Source: Front Neurosci. 2024 Feb 1;18:1324669. doi: 10.3389/fnins.2024.1324669 (PMC10867132; doi:10.3389/fnins.2024.1324669)
Supplement: Supplementary file 1 [file Data_Sheet_1.PDF]

Supplementary Material 1

From imaging to precision: Low cost and accurate determination of stereotactic coordinates for brain surgery *Sapajus apella* using MRI

Laís Resque Russo Pedrosa[1], Leon C. P. Leal[1,2], José Augusto P. C. Muniz[1,2], Caio de Oliveira Bastos[1], Bruno D. Gomes[1], Lane V. Krejcová[1]*

[1] Institute of Biological Sciences, Federal University of Pará, Belém, Pará State, Brazil.

[2] National Primate Center, Institute Evandro Chagas, Ananindeua, Pará State, Brazil.

***Correspondence:** Corresponding Author: lane@ufpa.br

1. SUPPLEMENTARY DATA

1.1 Steps for selecting the whole brain and surgical targets were as follows:

(1) Brain Volume Rendering and Calculation

MRI volume rendering was performed using the "Segment Editor" module. Brain volume was manually delineated by skull-stripping, with parts of the olfactory bulb and optic chiasms also being removed. This brain volume rendering serves as a spatial reference for MRI anatomical landmarks, which are essential for determining stereotaxic planes. Additionally, brain volume was quantified from the volume rendering in cubic millimeters (mm^3) using the "Segment Statistics" module.

(2) Anatomical Landmarks Positioning

Anatomical landmarks were identified according to MRI using the "Markups" module. Two landmarks were placed at the bilateral acoustic meatus (F1, F2), aligning with the interaural line. Another two landmarks were positioned at the bilateral infraorbital foramen (F3, F4).

(3) Stereotaxic Alignment

To simulate actual stereotaxic positioning, the alignment of the three stereotaxic planes was performed using the "Transforms" module. This alignment, based on MRI and brain volume rendering, established a new coordinate representing the stereotaxic origin.

(4) Translating Stereotaxic Origin to 3D Slicer Origin

A new transformation was created with the "Transforms" module. The inverse values of the origin coordinates were inputted in the translation box for left-right (LR), posteroanterior (AP), and inferior-superior (IS) axes to reset the 3D Slicer origin to zero.

(5) Fiducials Surgery Planning

Individual fiducial surgery planning involved eight surgical targets within the basal ganglia structures, unilateral in approach. Four fiducials were placed on the dominant motor side of the substantia nigra, with an additional four along the striatum. Each fiducial was located using the "Markups" module with specific LR, AP, and IS coordinates in millimeters.

(6) Validation

Various control fiducials were used for method validation. These included the anterior horns of the left and right sides (A1, A2), the posterior horns of the left and right sides (P1, P2), and the splenium and genu of the corpus callosum (S, G).

See the guide for processing MRI using Slicer 3D software in Supplementary Material 3

2. SUPPLEMENTARY TABLES AND FIGURES

1.1 Tables

Table 1 – Studies using *Sapajus apella* as an animal model in neuroscience over the last 20 years.

Year	Findings
2003	Cognition - 1 (McGonigle et al., 2003)
	Visual system - 1 (Jacobs and Deegan II, 2003)
	Poliovirus - 1 (Ida-Hosonuma et al., 2003)
2004	Mophorlogy - 1 (Horta-Júnior et al., 2004)
2005	Visual system - 4 (Gomes et al., 2005;Jacobs and Deegan, 2005;Saito et al., 2005a;Saito et al., 2005b)

2007	Morphophysiology - 1 (Pinato et al., 2007)
	Behaviour – 1 (Tavares et al., 2007)
	Motricity - 1 (Padberg et al., 2007)
2009	Morphophysiology -2 (Dum et al., 2009;Phillips et al., 2009)
2010	Morphophysiology -1 (Bostan et al., 2010)
2011	Visual system -1 (Ito et al., 2011)
	Pharmacology - 1 (Cavalcante et al., 2011)
	Behaviour - 2 (Brosnan et al., 2011;Fragaszy et al., 2011)
	Morphophysiology – 1 (Maior et al., 2011)
2012	Morphophisiology - 1 (Phillips and Sherwood, 2012)
2013	Morphophisiology – 1 (Aversi-Ferreira et al., 2013)
2014	Behaviour – 3 (Amici et al., 2014;Mayer et al., 2014;Saletti et al., 2014)
	Anatomy –1 (Aversi-Ferreira et al., 2014)
	Morphophisiology – 1 (Finlay et al., 2014)
2015	Morphophisiology – 3 (Borges et al., 2015;Charvet et al., 2015;Vasconcelos Braz et al., 2015)
	Pharmacology – 2 (Borges et al., 2015;Bowler et al., 2015)
2016	Cognition – 2 (Tecwyn et al., 2017;Truppa et al., 2017)
	Memory – 1 (Truppa et al., 2016)
	Morphophisiology – 4 (Hamadjida et al., 2016;Ohbayashi et al., 2016;Quessy et al., 2016;Lucarelli et al., 2017)

	Motricity – 1 (Dea et al., 2016)
2017	Visual System – 3 (Lucarelli et al., 2017;Stephenson et al., 2017;Truppa et al., 2017)
	Pharmacology – 2 (Lévesque et al., 2017;Lucarelli et al., 2017)
2018	Behaviour – 1 (Benítez et al., 2018)
2019	Behaviour – 2 (Broihanne et al., 2019;Smith et al., 2019)
2020	Cognition – 1 (Hirel et al., 2020)
	Behaviour – 2 (Heuberger et al., 2020;Trapanese et al., 2020)
	Morphophysiology – 1 (Ohbayashi, 2020)
2021	Cognition – 1 (Jordan et al., 2021)
	Morphophysiology – 1 (Hecht et al., 2021)
	Behaviour – 1 (Robinson et al., 2021)
2022	Behaviour – 2 (Roig et al., 2022;Trapanese et al., 2022)
	Mophophisiology – 2 (Lucore et al., 2022;Watson et al., 2022)
	Cognition – 2 (Miss et al., 2022;Sosnowski et al., 2022)
2023	Behaviour – 2 (Ciacci et al., 2023;Daoudi-Simison et al., 2023)
	Morphophysiology – 2 (Reilly et al., 2023;Sosnowski et al., 2023)

Table 2 – Mean and standard deviation (SD) error for anterior-posterior, dorsal-ventral, and medio-lateral axes in each animal.

ANTERIOR-POSTERIOR							
**AMASA**	**AMAXD**	**AMBBH**	**AMBCL**	**AMBEG**	**AMBEN**	**MEAN**	**SD**
0.0259	0.0182	0.0268	0.0107	0.0074	0.0313	0.02	0.01
**DORSAL-VENTRAL**							
**AMASA**	**AMAXD**	**AMBBH**	**AMBCL**	**AMBEG**	**AMBEN**	**MEAN**	**SD**
0.0782	0.021	0.0753	0.0239	0.0217	0.311	0.09	0.1
**MEDIO-LATERAL**							
**AMASA**	**AMAXD**	**AMBBH**	**AMBCL**	**AMBEG**	**AMBEN**	**MEAN**	**SD**
0.0107	0.012	0.0163	0.0177	0.0129	0.0087	0.013	0.003

2.2 Figures

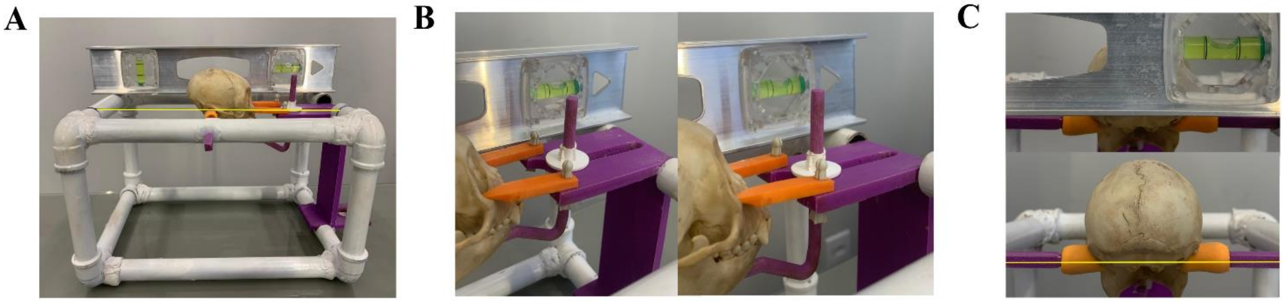

Figure S1. Demonstration of aligment to the apparatus using a measurement tools. (A) alignment of Frankfurt plane. (B) The stereotactic apparatus design allows the adjustment of the orbital and mouth adaptor moving the support system. (C) Interaural ear bars alignment.

References from Table 1

Amici, F., Visalberghi, E., and Call, J. (2014). Lack of prosociality in great apes, capuchin monkeys and spider monkeys: convergent evidence from two different food distribution tasks. *Proceedings of the Royal Society B: Biological Sciences* 281**,** 20141699.

Aversi-Ferreira, R.A., De Abreu, T., Pfrimer, G.A., Silva, S.F., Ziermann, J.M., Carneiro-E-Silva, F.O., Tomaz, C., Tavares, M.C.H., Maior, R.S., and Aversi-Ferreira, T.A. (2013). Comparative

anatomy of the hind limb vessels of the bearded capuchins (Sapajus libidinosus) with apes, baboons, and Cebus capucinus: with comments on the vessels' role in bipedalism. *BioMed Research International* 2013.

Aversi-Ferreira, R.A., Maior, R.S., Aziz, A., Ziermann, J.M., Nishijo, H., Tomaz, C., Tavares, M.C.H., and Aversi-Ferreira, T.A. (2014). Anatomical analysis of thumb opponency movement in the capuchin monkey (Sapajus sp). *PloS one* 9**,** e87288.

Benítez, M.E., Sosnowski, M.J., Tomeo, O.B., and Brosnan, S.F. (2018). Urinary oxytocin in capuchin monkeys: Validation and the influence of social behavior. *American Journal of Primatology* 80**,** e22877.

Borges, K.C.M., Nishijo, H., Aversi-Ferreira, T.A., Ferreira, J.R., and Caixeta, L.F. (2015). Anatomical study of intrahemispheric association fibers in the brains of capuchin monkeys (Sapajus sp.). *BioMed Research International* 2015.

Bostan, A.C., Dum, R.P., and Strick, P.L. (2010). The basal ganglia communicate with the cerebellum. *Proceedings of the national academy of sciences* 107**,** 8452-8456.

Bowler, M., Messer, E.J., Claidière, N., and Whiten, A. (2015). Mutual medication in capuchin monkeys–Social anointing improves coverage of topically applied anti-parasite medicines. *Scientific Reports* 5**,** 15030.

Broihanne, M.-H., Romain, A., Call, J., Thierry, B., Wascher, C.A., De Marco, A., Verrier, D., and Dufour, V. (2019). Monkeys (Sapajus apella and Macaca tonkeana) and great apes (Gorilla gorilla, Pongo abelii, Pan paniscus, and Pan troglodytes) play for the highest bid. *Journal of Comparative Psychology* 133**,** 301.

Brosnan, S.F., Parrish, A., Beran, M.J., Flemming, T., Heimbauer, L., Talbot, C.F., Lambeth, S.P., Schapiro, S.J., and Wilson, B.J. (2011). Responses to the Assurance game in monkeys, apes, and humans using equivalent procedures. *Proceedings of the National Academy of Sciences* 108**,** 3442-3447.

Cavalcante, J.C., Cândido, P.L., Sita, L.V., Do Nascimento Jr, E.S., De Souza Cavalcante, J., De Oliveira Costa, M.S.M., Bittencourt, J.C., and Elias, C.F. (2011). Comparative distribution of cocaine-and amphetamine-regulated transcript (CART) in the hypothalamus of the capuchin monkey (Cebus apella) and the common marmoset (Callithrix jacchus). *Brain research* 1425**,** 47-61.

Charvet, C.J., Cahalane, D.J., and Finlay, B.L. (2015). Systematic, cross-cortex variation in neuron numbers in rodents and primates. *Cerebral Cortex* 25**,** 147-160.

Ciacci, F., Mayerhoff, S., De Petrillo, F., Gastaldi, S., Brosnan, S.F., and Addessi, E. (2023). State-dependent risky choices in primates: Variation in energy budget does not affect tufted capuchin monkeys'(Sapajus spp.) risky choices. *American Journal of Primatology* 85**,** e23542.

Daoudi-Simison, S., O'sullivan, E., Moat, G., Lee, P.C., and Buchanan-Smith, H.M. (2023). Do mixed-species groups of capuchin (Sapajus apella) and squirrel monkeys (Saimiri sciureus) synchronize their behaviour? *Philosophical Transactions of the Royal Society B* 378**,** 20220111.

Dea, M., Hamadjida, A., Elgbeili, G., Quessy, S., and Dancause, N. (2016). Different patterns of cortical inputs to subregions of the primary motor cortex hand representation in Cebus apella. *Cerebral cortex* 26**,** 1747-1761.

Dum, R.P., Levinthal, D.J., and Strick, P.L. (2009). The spinothalamic system targets motor and sensory areas in the cerebral cortex of monkeys. *Journal of Neuroscience* 29**,** 14223-14235.

Finlay, B.L., Charvet, C.J., Bastille, I., Cheung, D.T., Muniz, J.a.P., and De Lima Silveira, L.C. (2014). Scaling the primate lateral geniculate nucleus: niche and neurodevelopment in the regulation of magnocellular and parvocellular cell number and nucleus volume. *Journal of Comparative Neurology* 522**,** 1839-1857.

Fragaszy, D.M., Stone, B.W., Scott, N.M., and Menzel, C. (2011). How tufted capuchin monkeys (Cebus apella spp) and common chimpanzees (Pan troglodytes) align objects to surfaces: Insights into spatial reasoning and implications for tool use. *American Journal of Primatology* 73**,** 1012-1030.

Gomes, Ú.R., Pessoa, D.M., Suganuma, E., Tomaz, C., and Pessoa, V.F. (2005). Influence of stimuli size on color discrimination in capuchin monkeys. *American Journal of Primatology: Official Journal of the American Society of Primatologists* 67**,** 437-446.

Hamadjida, A., Dea, M., Deffeyes, J., Quessy, S., and Dancause, N. (2016). Parallel cortical networks formed by modular organization of primary motor cortex outputs. *Current Biology* 26**,** 1737-1743.

Hecht, E.E., Reilly, O.T., Benítez, M.E., Phillips, K.A., and Brosnan, S.F. (2021). Sex differences in the brains of capuchin monkeys (Sapajus [Cebus] apella). *Journal of Comparative Neurology* 529**,** 327-339.

Heuberger, B., Paukner, A., Wooddell, L.J., Kasman, M., and Hammond, R.A. (2020). The role of novelty and fat and sugar concentration in food selection by captive tufted capuchins (Sapajus apella). *American journal of primatology* 82**,** e23165.

Hirel, M., Thiriau, C., Roho, I., and Meunier, H. (2020). Are monkeys able to discriminate appearance from reality? *Cognition* 196**,** 104123.

Horta-Júnior, J., Tamega, O., and Cruz-Rizzolo, R. (2004). Cytoarchitecture and musculotopic organization of the facial motor nucleus in Cebus apella monkey. *Journal of anatomy* 204**,** 175-190.

Ida-Hosonuma, M., Sasaki, Y., Toyoda, H., Nomoto, A., Gotoh, O., Yonekawa, H., and Koike, S. (2003). Host range of poliovirus is restricted to simians because of a rapid sequence change of the poliovirus receptor gene during evolution. *Archives of virology* 148**,** 29-44.

Ito, J., Maldonado, P., Singer, W., and Grün, S. (2011). Saccade-related modulations of neuronal excitability support synchrony of visually elicited spikes. *Cerebral cortex* 21**,** 2482-2497.

Jacobs, G.H., and Deegan Ii, J.F. (2003). Cone pigment variations in four genera of New World monkeys. *Vision research* 43**,** 227-236.

Jacobs, G.H., and Deegan, J.F. (2005). Polymorphic New World monkeys with more than three M/L cone types. *JOSA A* 22**,** 2072-2080.

Jordan, E.J., Völter, C.J., and Seed, A.M. (2021). Do capuchin monkeys (Sapajus apella) use exploration to form intuitions about physical properties? *Cognitive Neuropsychology* 38**,** 531-543.

Lévesque, C., Hernandez, G., Mahmoudi, S., Calon, F., Gasparini, F., Gomez-Mancilla, B., Blanchet, P.J., and Lévesque, D. (2017). Deficient striatal adaptation in aminergic and glutamatergic

neurotransmission is associated with tardive dyskinesia in non-human primates exposed to antipsychotic drugs. *Neuroscience* 361**,** 43-57.

Lucarelli, M., Visalberghi, E., Adriani, W., Addessi, E., Pierandrei, S., Manciocco, A., Zoratto, F., Tamellini, A., Vitale, A., and Laviola, G. (2017). Polymorphism of the 3′-UTR of the dopamine transporter gene (DAT) in New World monkeys. *Primates* 58**,** 169-178.

Lucore, J.M., Marshall, A.J., Brosnan, S.F., and Benítez, M.E. (2022). Validating urinary neopterin as a biomarker of immune response in captive and wild capuchin monkeys. *Frontiers in Veterinary Science* 9**,** 918036.

Maior, R.S., Hori, E., Barros, M., Teixeira, D.S., Tavares, M.C.H., Ono, T., Nishijo, H., and Tomaz, C. (2011). Superior colliculus lesions impair threat responsiveness in infant capuchin monkeys. *Neuroscience letters* 504**,** 257-260.

Mayer, C., Call, J., Albiach-Serrano, A., Visalberghi, E., Sabbatini, G., and Seed, A. (2014). Abstract knowledge in the broken-string problem: evidence from nonhuman primates and pre-schoolers. *PLoS One* 9**,** e108597.

Mcgonigle, B., Chalmers, M., and Dickinson, A. (2003). Concurrent disjoint and reciprocal classification by Cebus apella in seriation tasks: Evidence for hierarchical organization. *Animal cognition* 6**,** 185-197.

Miss, F.M., Sadoughi, B., Meunier, H., and Burkart, J.M. (2022). Individual differences in co-representation in three monkey species (Callithrix jacchus, Sapajus apella and Macaca tonkeana) in the joint Simon task: The role of social factors and inhibitory control. *Animal Cognition* 25**,** 1399-1415.

Ohbayashi, M. (2020). Inhibition of protein synthesis in M1 of monkeys disrupts performance of sequential movements guided by memory. *Elife* 9**,** e53038.

Ohbayashi, M., Picard, N., and Strick, P.L. (2016). Inactivation of the dorsal premotor area disrupts internally generated, but not visually guided, sequential movements. *Journal of Neuroscience* 36**,** 1971-1976.

Padberg, J., Franca, J.G., Cooke, D.F., Soares, J.G., Rosa, M.G., Fiorani, M., Gattass, R., and Krubitzer, L. (2007). Parallel evolution of cortical areas involved in skilled hand use. *Journal of Neuroscience* 27**,** 10106-10115.

Phillips, K., Kapfenberger, N., and Hopkins, W. (2009). A comparative study of corpus callosum size and signal intensity in capuchin monkeys (Cebus apella) and chimpanzees (Pan troglodytes). *Neuroscience* 159**,** 1119-1125.

Phillips, K.A., and Sherwood, C.C. (2012). Age-related differences in corpus callosum area of capuchin monkeys. *Neuroscience* 202**,** 202-208.

Pinato, L., Allemandi, W., Abe, L.K., Frazão, R., Cruz-Rizzolo, R.J., Cavalcante, J.S., Costa, M.S., and Nogueira, M.I. (2007). A comparative study of cytoarchitecture and serotonergic afferents in the suprachiasmatic nucleus of primates (Cebus apella and Callithrix jacchus) and rats (Wistar and Long Evans strains). *Brain research* 1149**,** 101-110.

Quessy, S., Côté, S.L., Hamadjida, A., Deffeyes, J., and Dancause, N. (2016). Modulatory effects of the ipsi and contralateral ventral premotor cortex (PMv) on the primary motor cortex (M1) outputs to intrinsic hand and forearm muscles in Cebus apella. *Cerebral cortex* 26**,** 3905-3920.

Reilly, O.T., Brosnan, S.F., Benítez, M.E., Phillips, K.A., and Hecht, E.E. (2023). Sex differences in white matter tracts of capuchin monkey brains. *Journal of Comparative Neurology*.

Robinson, L.M., Martínez, M., Leverett, K.L., Rossettie, M.S., Wilson, B.J., and Brosnan, S.F. (2021). Anything for a cheerio: Brown capuchins (Sapajus [Cebus] apella) consistently coordinate in an Assurance Game for unequal payoffs. *American Journal of Primatology* 83**,** e23321.

Roig, A., Meunier, H., Poulingue, E., Marty, A., Thouvarecq, R., and Rivière, J. (2022). Is economic risk proneness in young children (Homo sapiens) driven by exploratory behavior? A comparison with capuchin monkeys (Sapajus apella). *Journal of Comparative Psychology* 136**,** 140.

Saito, A., Kawamura, S., Mikami, A., Ueno, Y., Hiramatsu, C., Koida, K., Fujita, K., Kuroshima, H., and Hasegawa, T. (2005a). Demonstration of a genotype–phenotype correlation in the polymorphic color vision of a non-callitrichine New World monkey, capuchin (Cebus apella). *American Journal of Primatology: Official Journal of the American Society of Primatologists* 67**,** 471-485.

Saito, A., Mikami, A., Kawamura, S., Ueno, Y., Hiramatsu, C., Widayati, K.A., Suryobroto, B., Teramoto, M., Mori, Y., and Nagano, K. (2005b). Advantage of dichromats over trichromats in discrimination of color-camouflaged stimuli in nonhuman primates. *American Journal of Primatology: Official Journal of the American Society of Primatologists* 67**,** 425-436.

Saletti, P.G., Maior, R.S., Hori, E., Almeida, R.M.D., Nishijo, H., and Tomaz, C. (2014). Whole-body prepulse inhibition protocol to test sensorymotor gating mechanisms in monkeys. *PLoS One* 9**,** e105551.

Smith, M.F., Leverett, K.L., Wilson, B.J., and Brosnan, S.F. (2019). Capuchin monkeys (Sapajus [Cebus] apella) play Nash equilibria in dynamic games, but their decisions are likely not influenced by oxytocin. *American Journal of Primatology* 81**,** e22973.

Sosnowski, M.J., Kano, F., and Brosnan, S.F. (2022). Oxytocin and social gaze during a dominance categorization task in tufted capuchin monkeys. *Frontiers in Psychology* 13**,** 977771.

Sosnowski, M.J., Reilly, O.T., Brosnan, S.F., and Benítez, M.E. (2023). Oxytocin increases during fur-rubbing regardless of level of social contact in tufted capuchin monkeys. *American Journal of Primatology***,** e23490.

Stephenson, A.R., Edler, M.K., Erwin, J.M., Jacobs, B., Hopkins, W.D., Hof, P.R., Sherwood, C.C., and Raghanti, M.A. (2017). Cholinergic innervation of the basal ganglia in humans and other anthropoid primates. *Journal of Comparative Neurology* 525**,** 319-332.

Tavares, M.C., Topic, B., Abreu, C., Waga, I., Gomes, Ú., Tomaz, C., and Mattern, C. (2007). Effects of intra-nasally administered testosterone on sexual proceptive behavior in female capuchin monkeys (Cebus apella). *Behavioural brain research* 179**,** 33-42.

Tecwyn, E.C., Denison, S., Messer, E.J., and Buchsbaum, D. (2017). Intuitive probabilistic inference in capuchin monkeys. *Animal cognition* 20**,** 243-256.

Trapanese, C., Bey, M., Tonachella, G., Meunier, H., and Masi, S. (2020). Prolonged care and cannibalism of infant corpse by relatives in semi-free-ranging capuchin monkeys. *Primates* 61**,** 41-47.

Trapanese, C., Meunier, H., and Masi, S. (2022). Do primates flexibly use spatio-temporal cues when foraging? *Quarterly Journal of Experimental Psychology* 75**,** 232-244.

Truppa, V., Carducci, P., De Simone, D.A., Bisazza, A., and De Lillo, C. (2017). Global/local processing of hierarchical visual stimuli in a conflict–choice task by capuchin monkeys (Sapajus spp.). *Animal cognition* 20**,** 347-357.

Truppa, V., De Simone, D.A., and De Lillo, C. (2016). Short-term memory effects on visual global/local processing in tufted capuchin monkeys (Sapajus spp.). *Journal of Comparative Psychology* 130**,** 162.

Vasconcelos Braz, S., Monge-Fuentes, V., Rodrigues Da Silva, J., Tomaz, C., Tavares, M.C., Pereira Garcia, M., Nair Báo, S., Paulino Lozzi, S., and Bentes De Azevedo, R. (2015). Morphological analysis of reticuloendothelial system in Capuchin Monkeys (Sapajus spp.) after meso-2, 3-dimercaptosuccinic acid (DMSA) coated magnetic nanoparticles administration. *PloS one* 10**,** e0140233.

Watson, C.M., Sherwood, C.C., and Phillips, K.A. (2022). Myelin characteristics of the corpus callosum in capuchin monkeys (Sapajus [Cebus] apella) across the lifespan. *Scientific Reports* 12**,** 8786.
