# Supplementary material for: From imaging to precision: low cost and accurate determination of stereotactic coordinates for brain surgery Sapajus apella using MRI
Source: Front Neurosci. 2024 Feb 1;18:1324669. doi: 10.3389/fnins.2024.1324669 (PMC10867132; doi:10.3389/fnins.2024.1324669)

Supplementary Material 3

From imaging to precision: Low cost and accurate determination of stereotactic coordinates for brain surgery *Sapajus apella* using MRI

Laís Resque Russo Pedrosa[1], Leon C. P. Leal[1,2], José Augusto P. C. Muniz[1,2], Caio de Oliveira Bastos[1], Bruno D. Gomes[1], Lane V. Krejcová[1]*

[1] Institute of Biological Sciences, Federal University of Pará, Belém, Pará State, Brazil.

[2] National Primate Center, Institute Evandro Chagas, Ananindeua, Pará State, Brazil.

***Correspondence:** Corresponding Author: lane@ufpa.br

Importing DICOM file to 3D Slicer

1. Loads a DICOM file into the scene (DATA) and saves it into the database. It can also be loaded and examined (DCM)

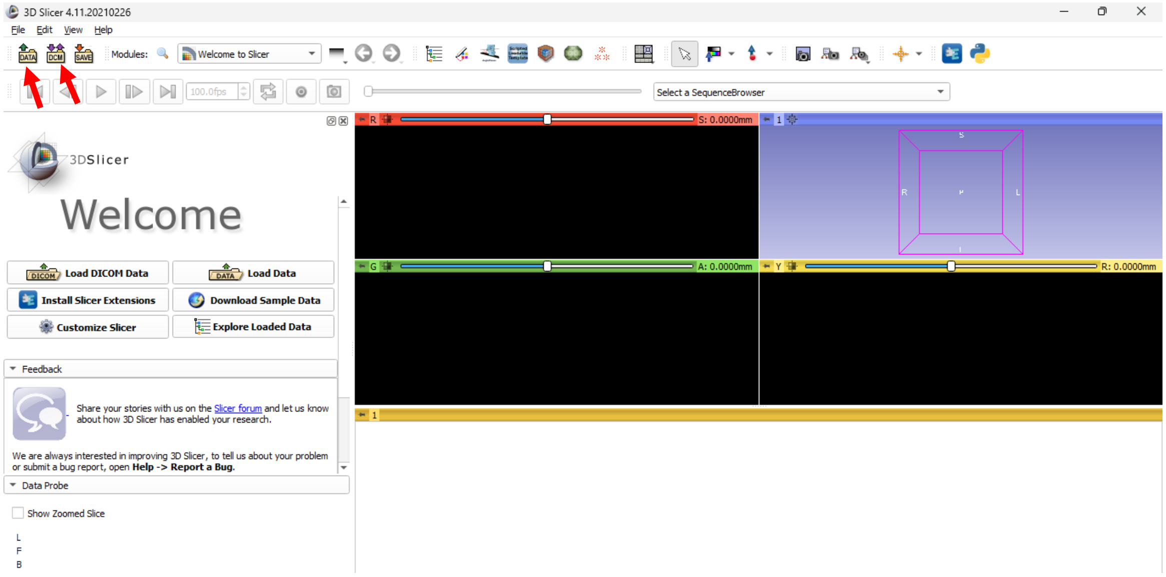

2. At data ( ), the full MRI sequence can be seen in widescreen showing volumetric sequence and axial/coronal/sagittal.

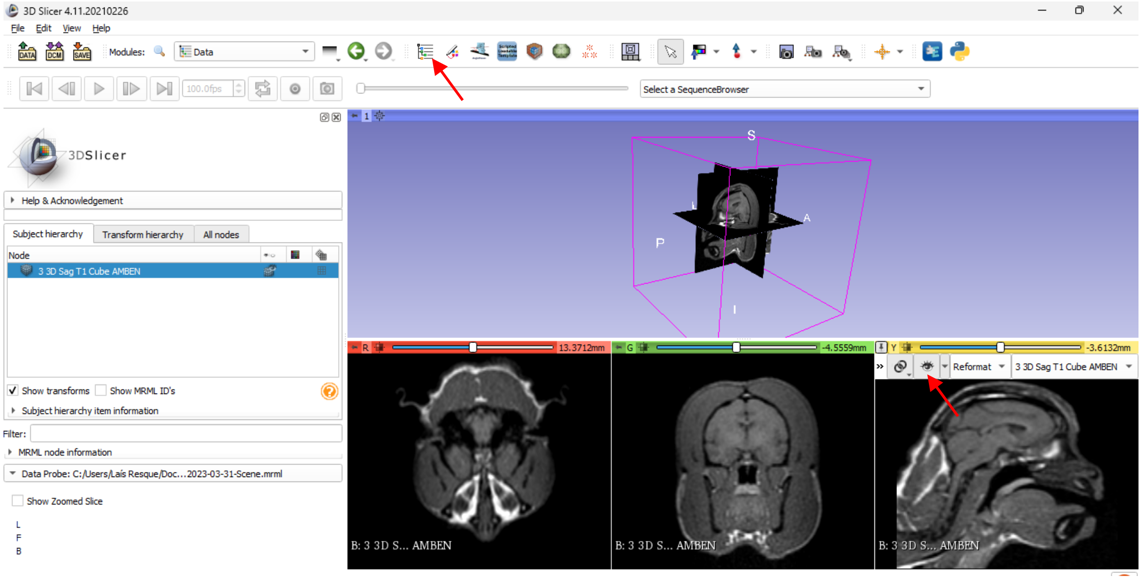

Rendering and parameters calculation

1. Using the Segment Editor module ( ), a new "segmentation" is created by selecting a "master volume" to enable editing. Two segmentations are recommended, head for stereotaxic alignment and brain for volume calculation.

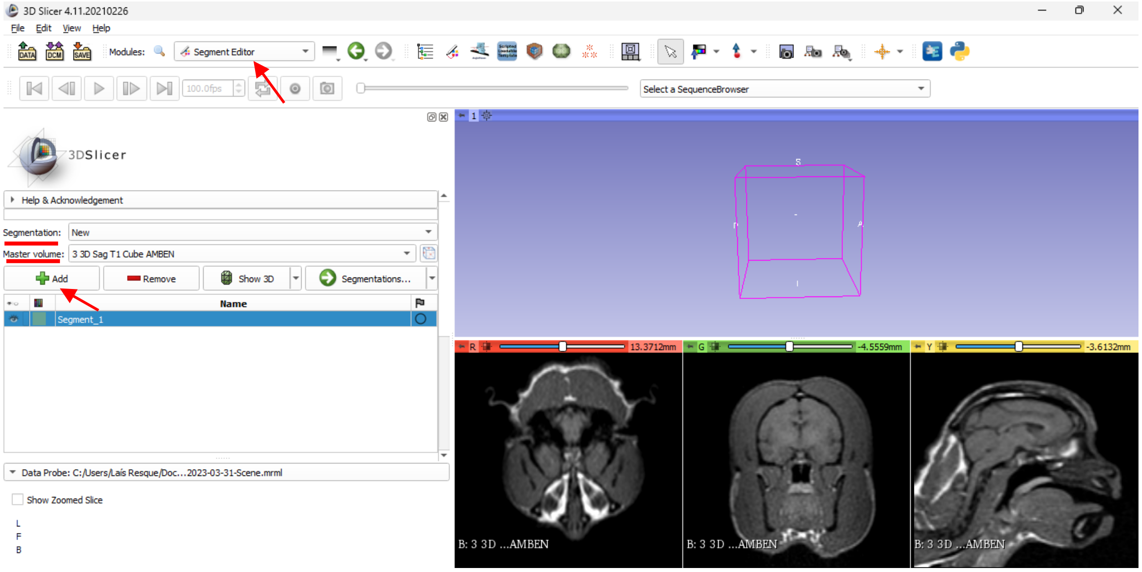

2. After segmentation, some effects tools will be available. Using “Threshold” ( ), the threshold range can be adjusted for setting the previewed segmentation and "Apply” to confirm the range just settled. The module "show 3D" shows the result.

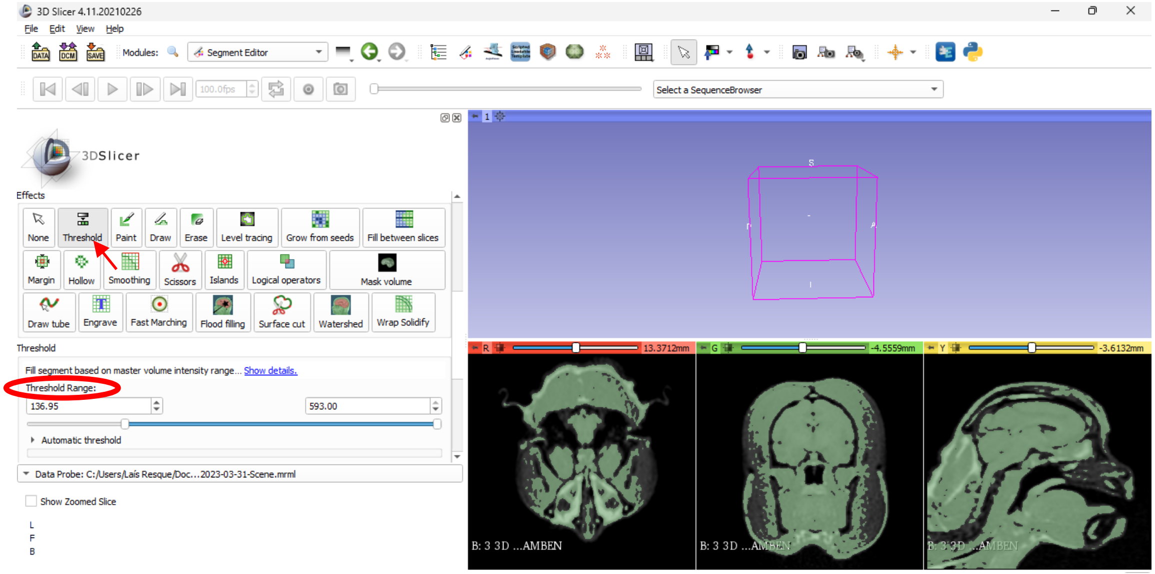

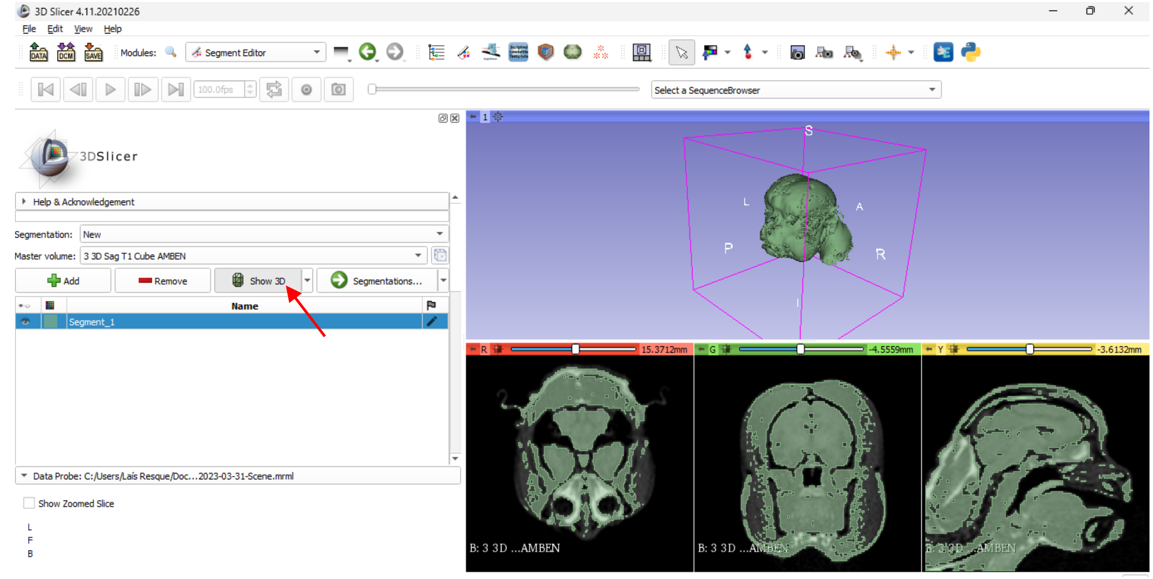

3. Using the scissors ( ), you select and cut out unwanted segments (left-button drag-and-drop).

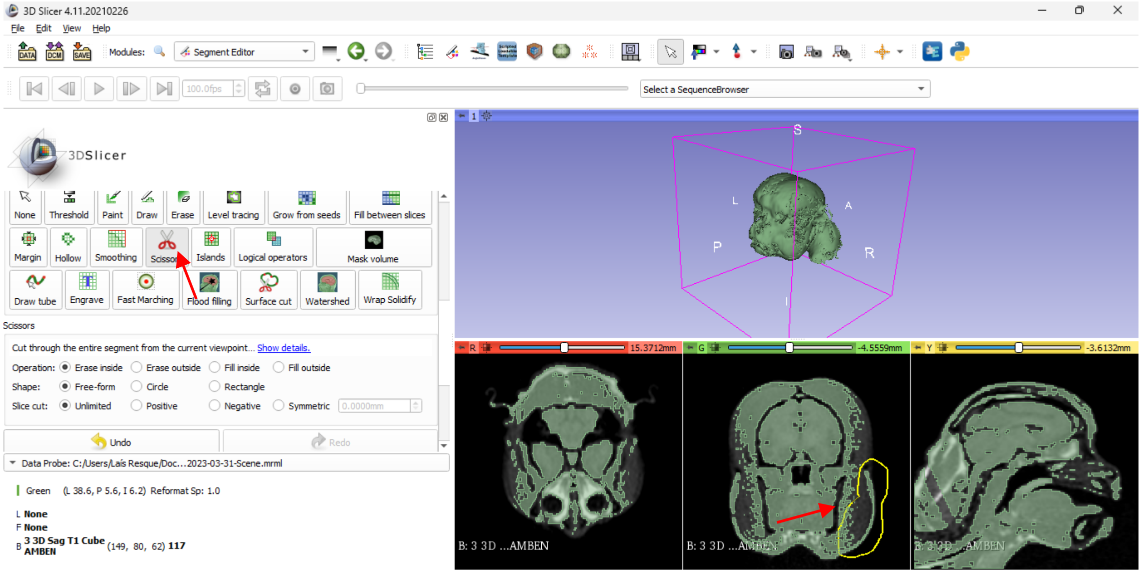

Head segmentation

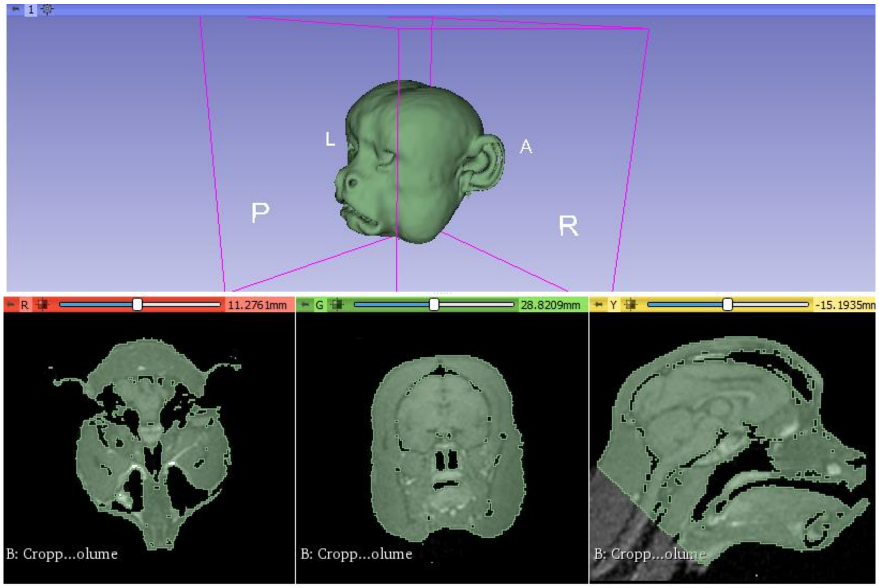

Brain segmentation

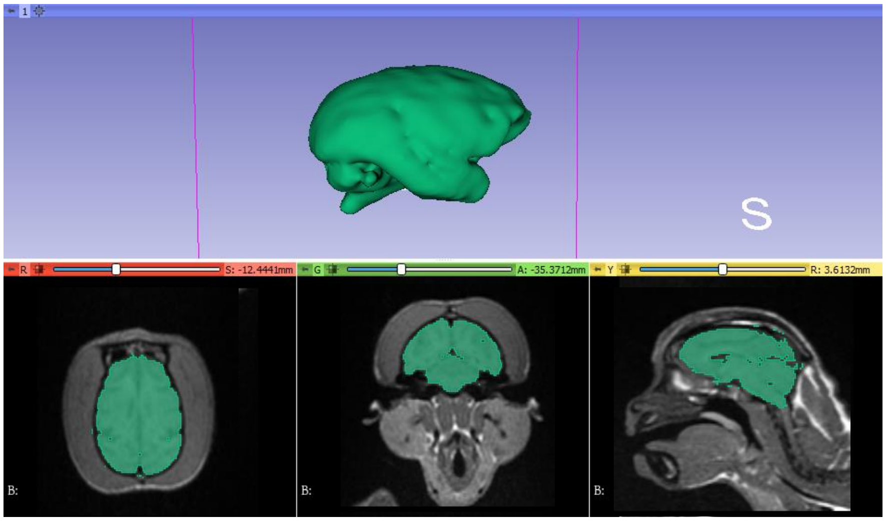

4. Using the "Segments statistics" module ( ) at the input menu, the created brain segmentation is selected followed by the selection of the MRI sequence as "scalar volume". After choosing "Apply", the fields provide the voxel count, and volume (in mm^3 and cm^3).

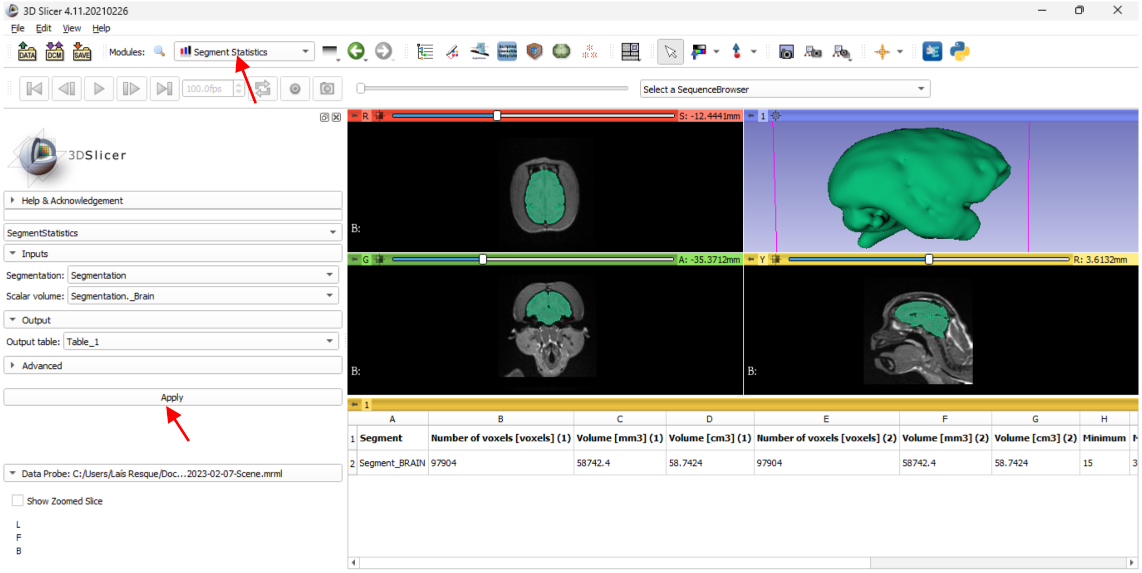

Anatomical landmark positioning

1. Using the Markups module ( ), groups of specialized fiducial markers can be created ( ). For adding markers in the same group, the "Create and Place" tool is selected ( ). The Shift ⇧ can be used to see the marker at all three planes for better positioning. At the "Display" tab, it is recommended to activate the 2D Display projection visibility.

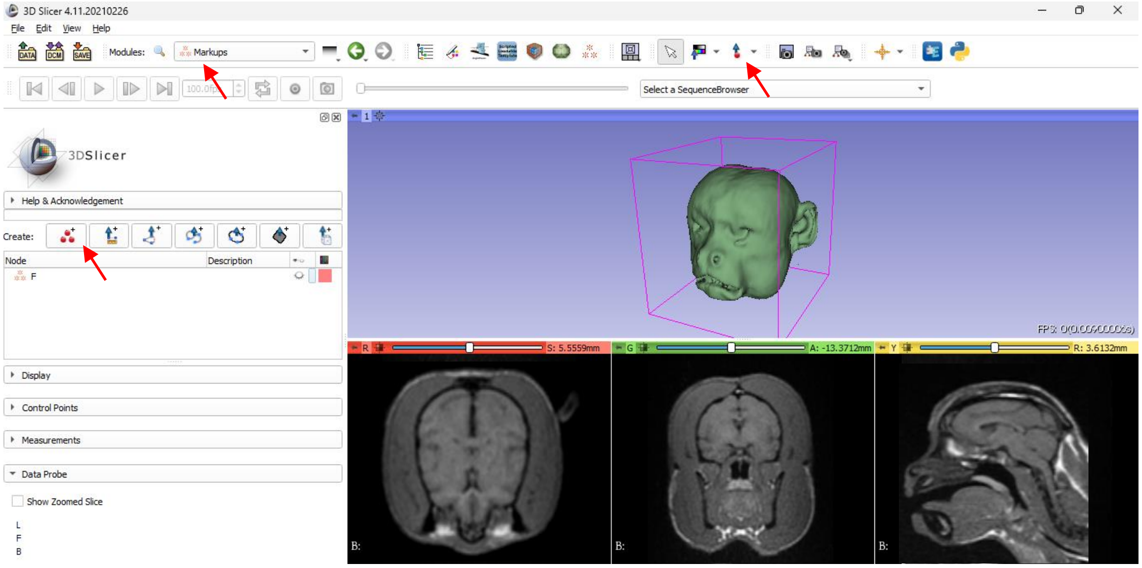

2. It is recommended to create two groups, the anatomical fiducial markers for stereotaxic alignment (see Figure 2), and the potential surgical target groups. At the "control points" tab, it is recommended to lock markups to prevent them from being moved by mistake.

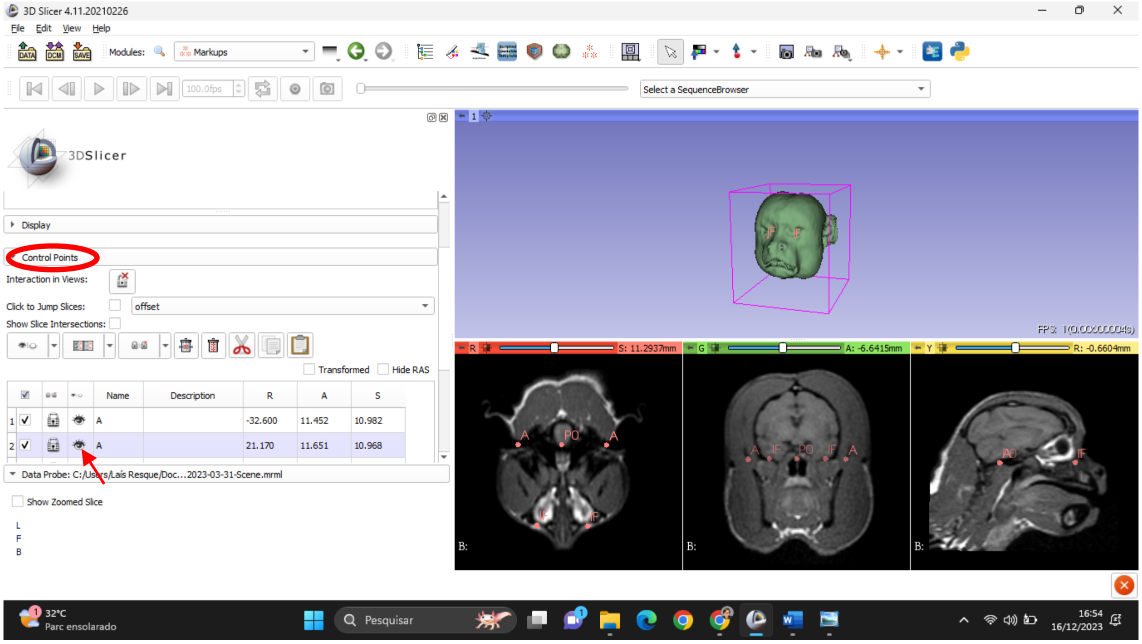

Stereotaxic alignment

1. Using the Transform module ( ), you can create and edit by choosing "Active transform". At the "Apply transform" tab, the MRI sequences and segmentations are selected to apply to the selected transformable nodes.

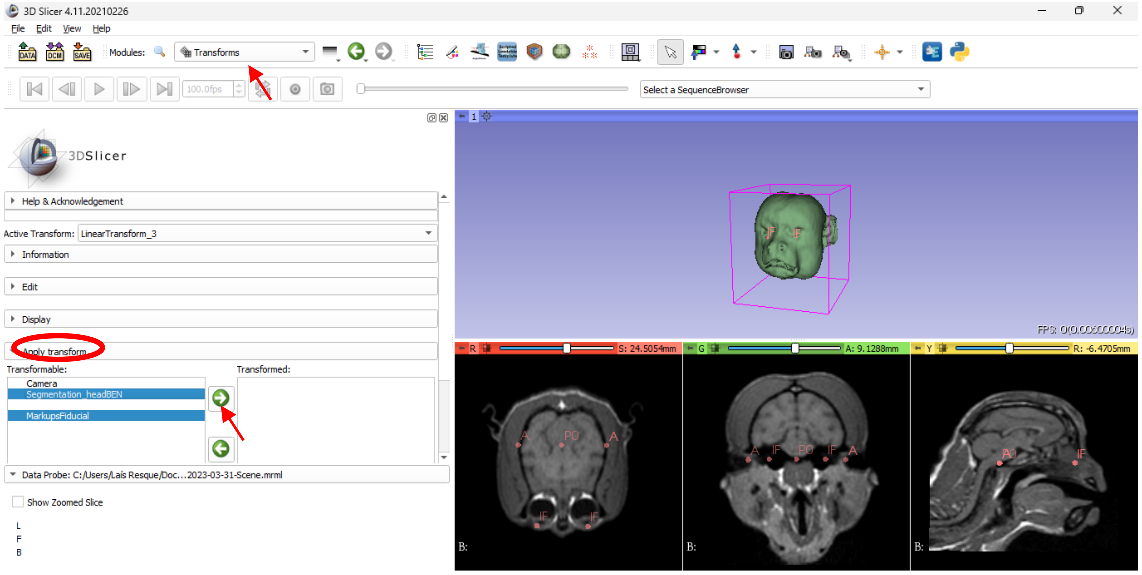

2. Using the rule tool ( ) as an alignment guide (it can be moved by pressing Shift ⇧), the "rotation" tab provides LR, PA, and IS rotations in degrees. (Suggestion LR= 90º, PA= 0º, IS= 180º).

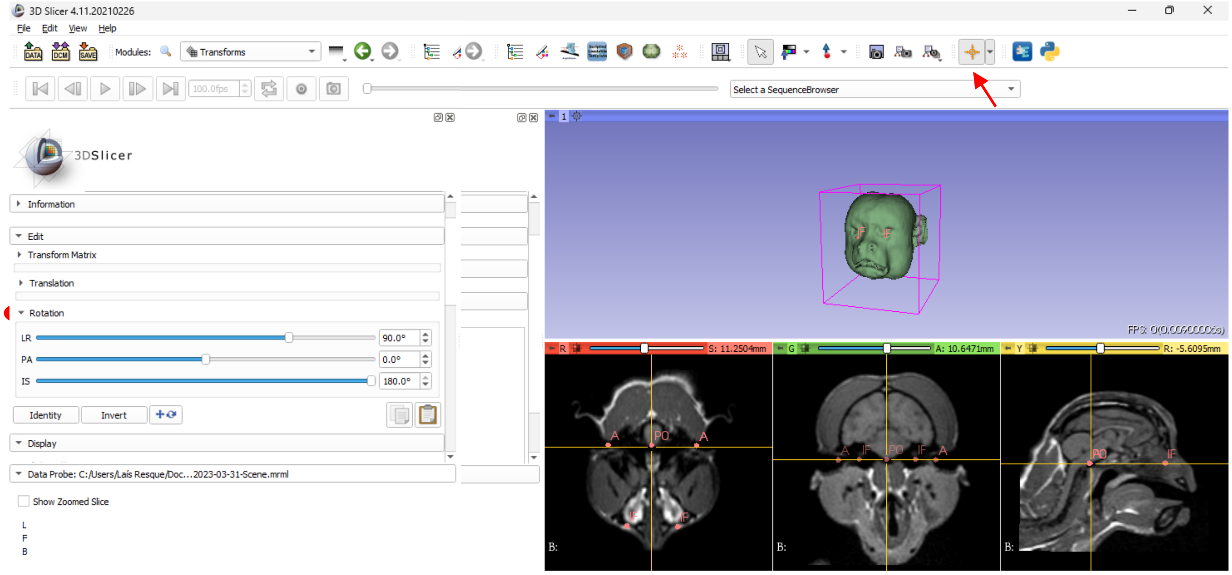

3. For alignment confirmation select "Harden transforms" at the "Apply transform" tab.

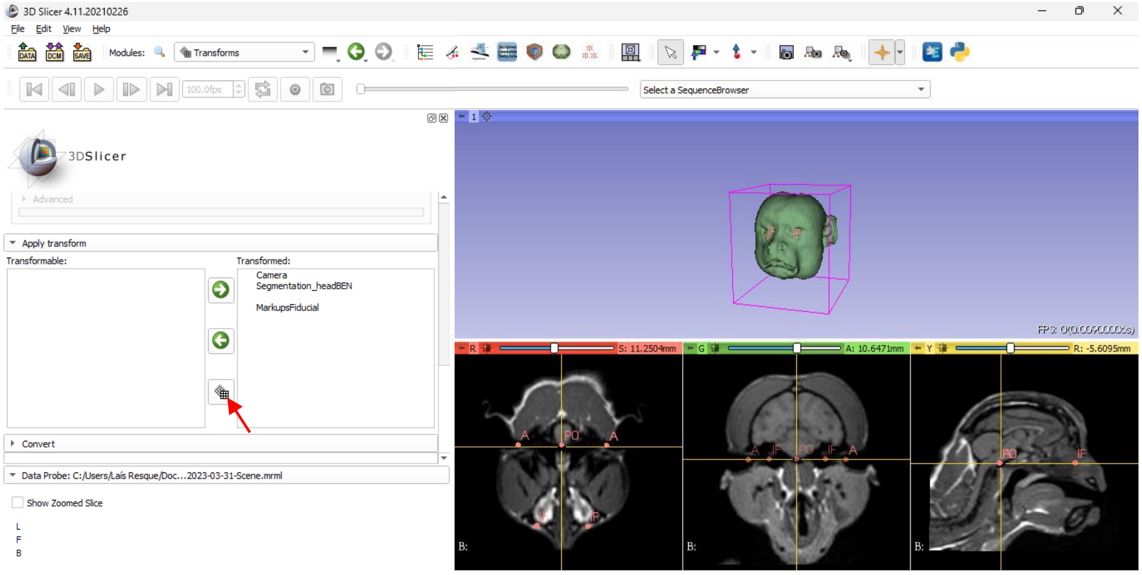

Translating stereotaxic origin to 3D Slicer origin

1. Going back to the markups module ( ), in the control points tab, the three values of stereotaxic origin (P0) can be seen.

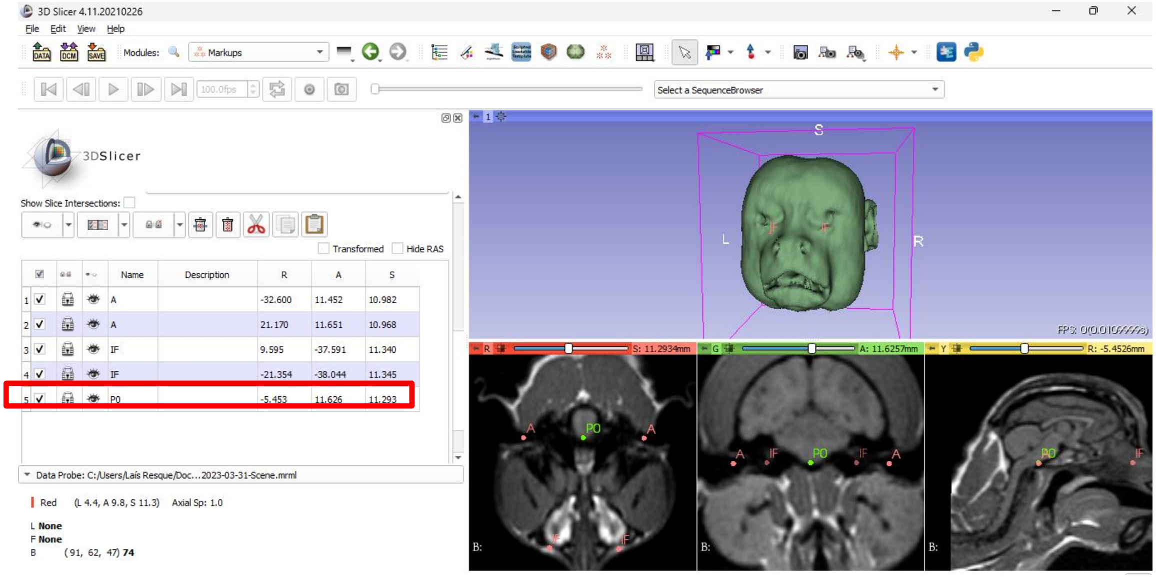

2. Using the Transform module ( ), you can align the MRI sequence. Select all the data in the transformation box. The translation value is the opposite where the stereotaxic origin is located (e.g. R: -5.453 → +5.453; A: +11.626 → -11.626; S: +11.293 → -11.293).

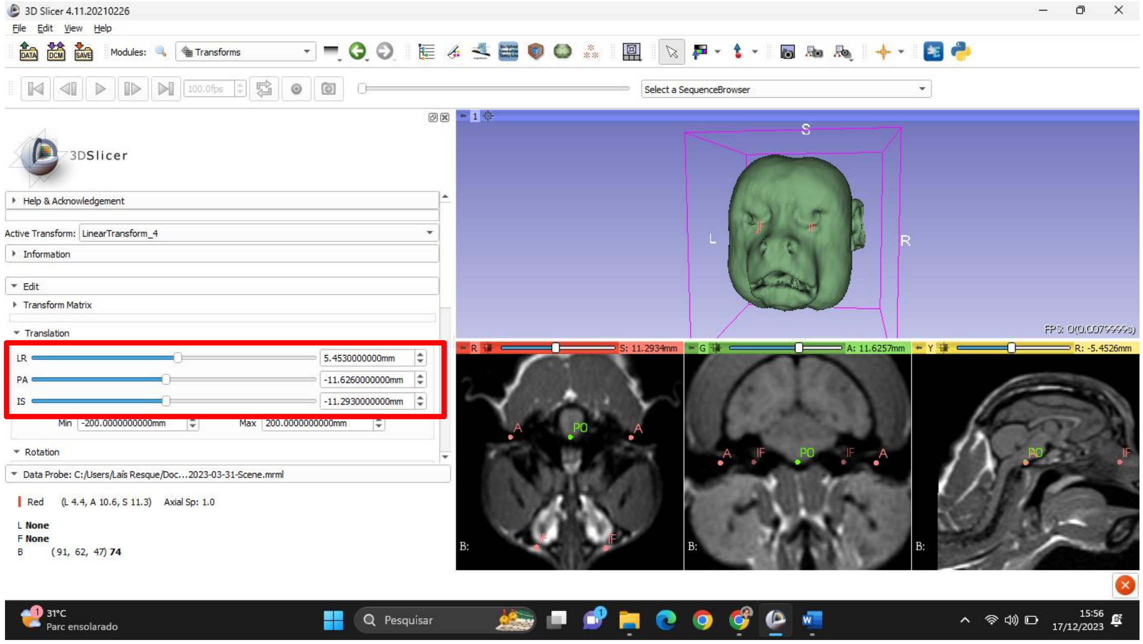

3. By inputting those numbers into the translation tab (Transform module), the P0 is converted to zero in the three axes.

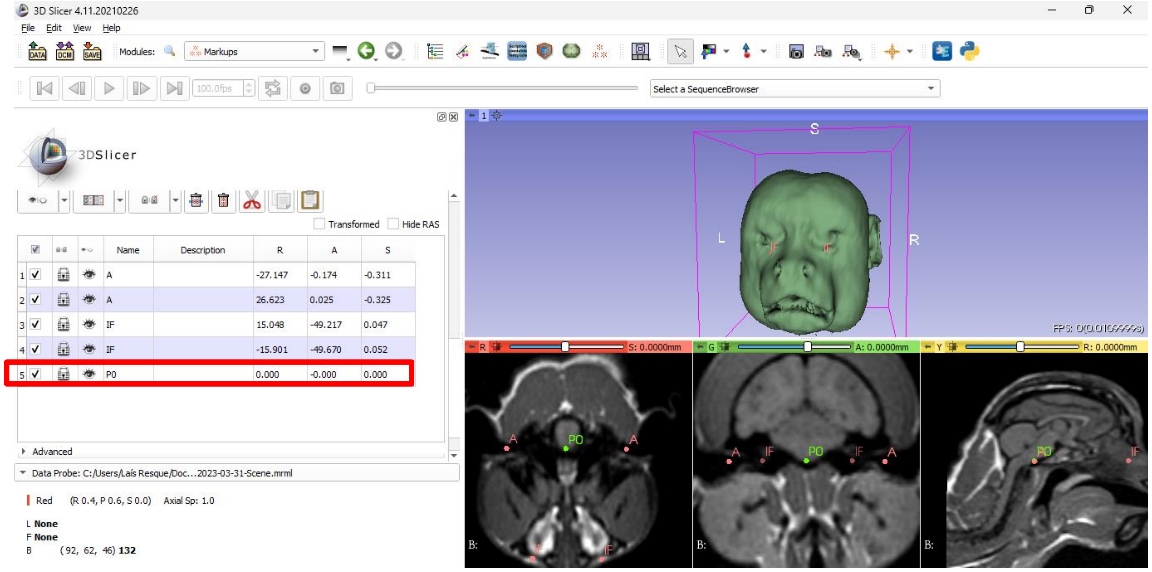

Fiducial surgery planning

1. Using the Markups module ( ), the potential surgery targets can be planned according to individual MRIs.

Example of potential surgery targets in substantia nigra (SN).

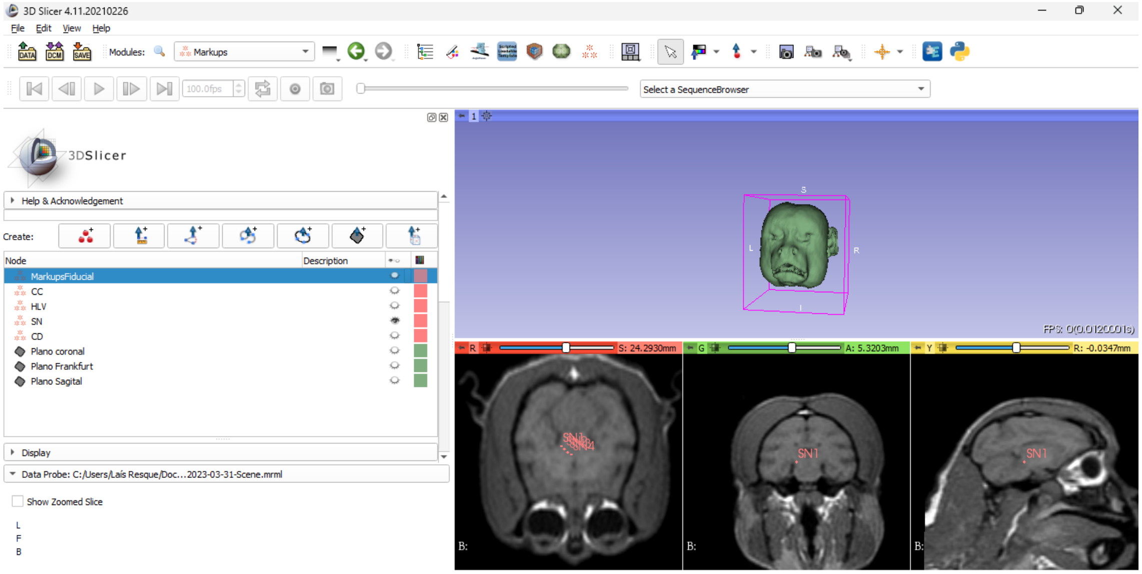

2. Our interest is the basal ganglia, so we distributed four equidistant fiducials markers in the substantia nigra, other four in the caudate nucleus ( ). Each fiducial planned was located in specific coordinates in IS (red), AP (green), and LR (yellow) in mm.

Another example of potential surgery targets the caudate nucleus (CD).

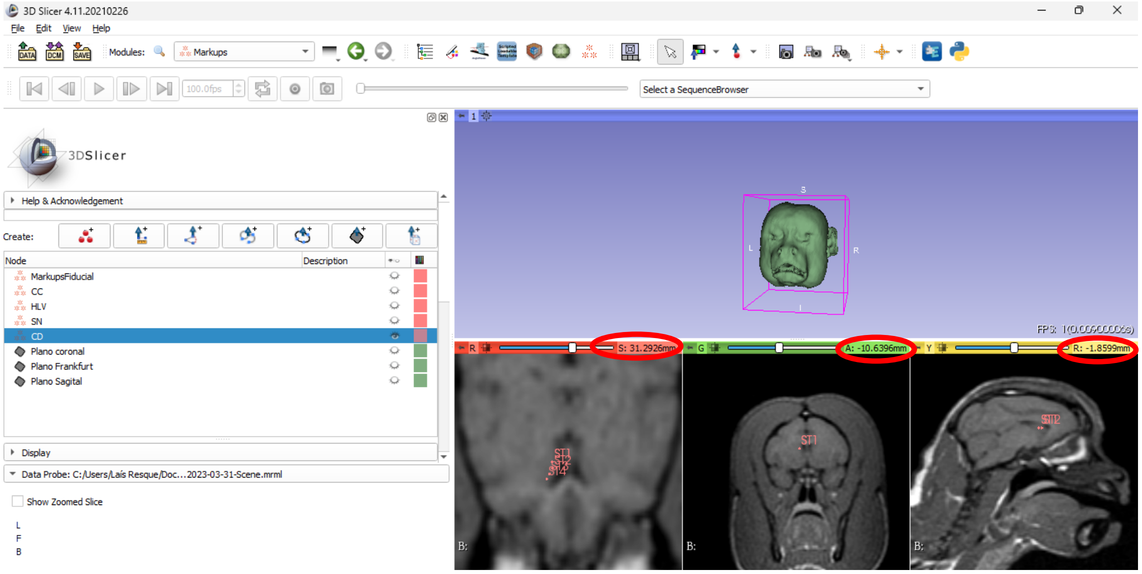

Validation

1. Using the Markups module ( ), we create a control fiducial markers group. Four bilateral anterior and posterior markers in the horns of lateral ventricles and two extremal corpus callosum structures (for creating a control fiducial group ; for adding more fiducial markers in the same group).

Example of control fiducial markers group in horns of lateral ventricles (HLV).

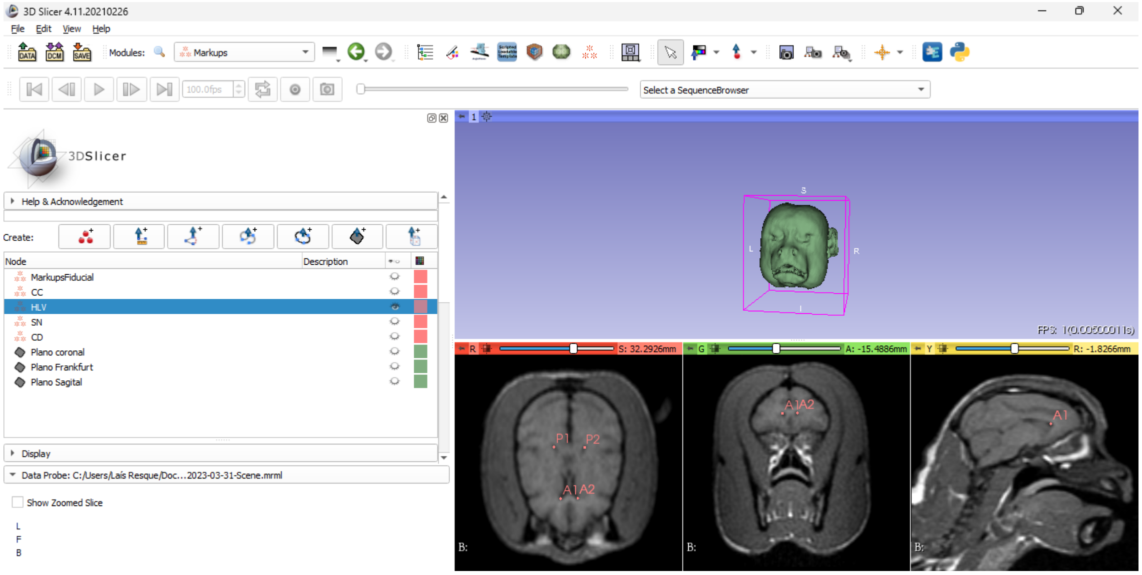

Visual confirmation of coordinates

1. Using the Markups module again, use the “Plane” tool ( ) to generate a plane that passes through the three points (markups) as depicted in this example:

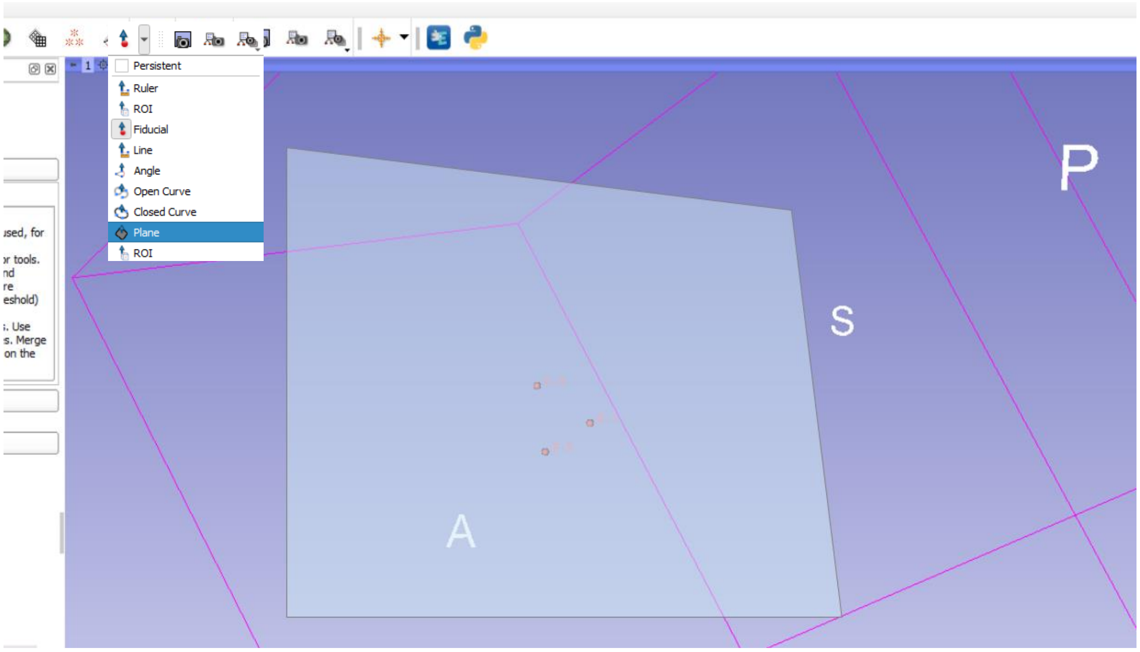

2. Each plane must be planned according to each axis (axial/coronal/sagittal). See also Figure 2.

Axial Plane (formed along the axial axis)

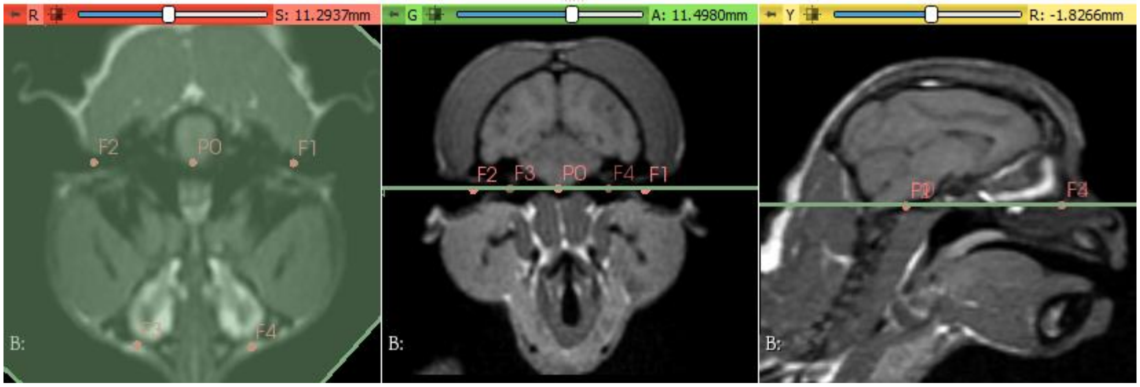

Coronal plane (formed along the coronal axis)

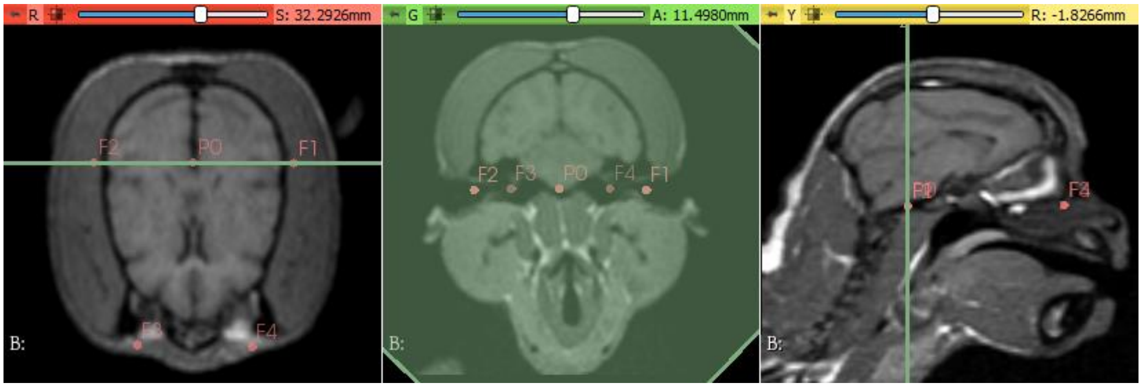

Sagittal plane (formed along the sagittal axis)

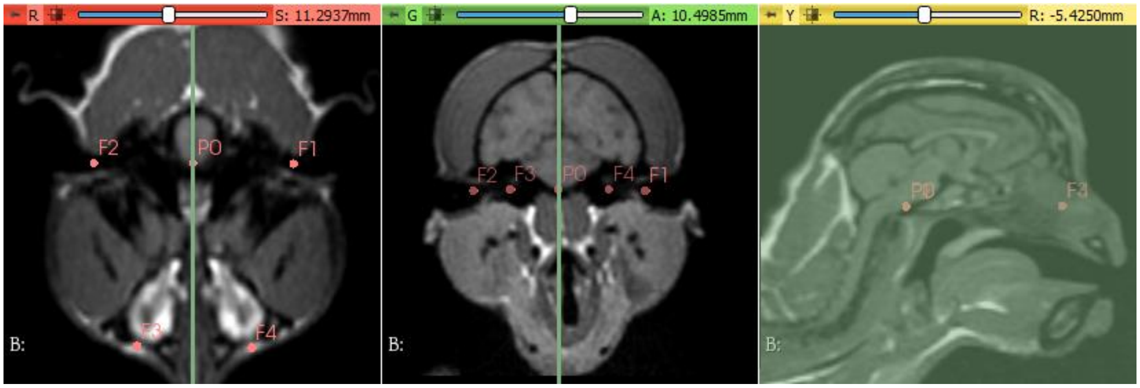

3. At each plane, potential surgery marker coordinates can be confirmed using a ruler (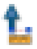). See also Figure 3C.

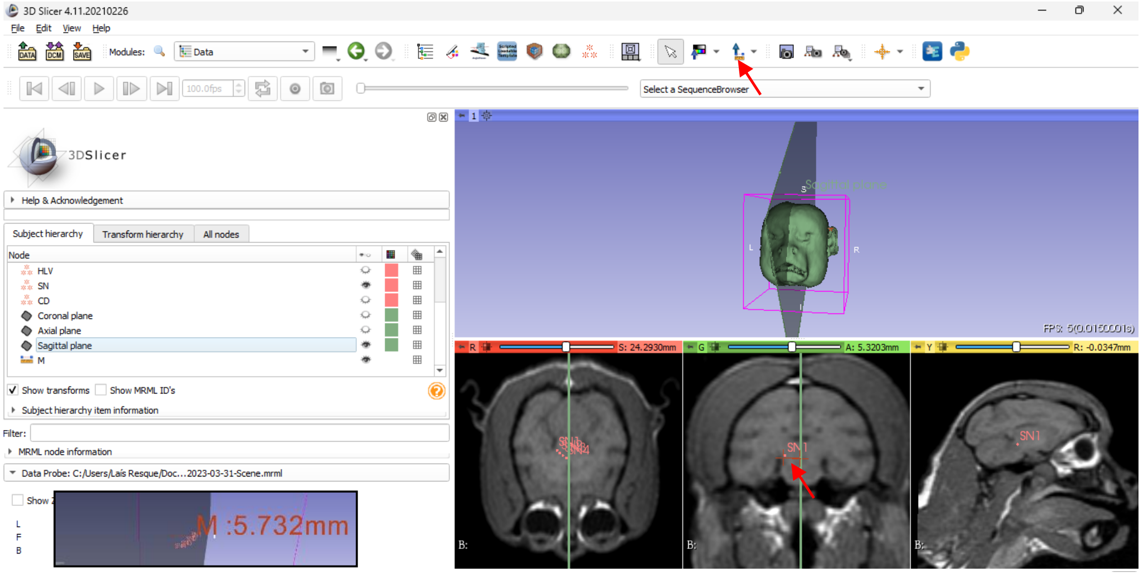

Supplement: Supplementary file 3 [file Data_Sheet_3.PDF]
